# Game theoretic research on strategies for medical carbon neutrality stakeholders under time delay effects

**DOI:** 10.3389/fpubh.2025.1640294

**Published:** 2025-09-09

**Authors:** Bo Xie, Xuyang Gao, Yingying Cheng, Muqing Niu, Shijia Liu

**Affiliations:** ^1^Business School and MBA Education Center of Henan University of Science and Technology, Luoyang, Henan, China; ^2^Henley Business School, University of Reading, Reading, United Kingdom; ^3^Affiliated First Hospital, Henan University of Science and Technology, Luoyang, Henan, China; ^4^School of Business Administration, Henan University of Economics and Law, Zhengzhou, Henan, China

**Keywords:** medical carbon neutrality, medical supply chain, public health policy, four-party evolutionary game, time delay

## Abstract

**Introduction:**

Carbon emissions from the healthcare sector have drawn increasing attention as global climate change intensifies. Achieving carbon neutrality in healthcare is vital for sustainable development, yet the complexity of stakeholder interactions and regulatory mechanisms creates substantial challenges.

**Methods:**

This study develops a dynamic evolutionary game model with time-delay effects to examine the strategic behaviors of four stakeholders: government, public hospitals, pharmaceutical enterprises, and the public. Stability analysis is applied to determine equilibrium strategies, while MATLAB simulations explore the impacts of regulatory, financial, and reputational parameters on system dynamics.

**Results:**

The findings reveal that government regulation is constrained by high costs and limited credibility, indicating the need to reduce costs and enhance credibility through market-based carbon trading and information-driven supervision. Public hospitals' weak compliance, driven by short-term profit motives, can be improved by strengthening financial incentives and penalties. Pharmaceutical enterprises' self-discipline is promoted by raising fines, reinforcing reputational incentives, and expanding public oversight channels. Public participation exerts a significant influence on carbon neutrality outcomes, and optimized online engagement mechanisms coupled with flexible compensation ratios are found to enhance effectiveness. Time delays accelerate system convergence but do not alter the evolutionary direction.

**Discussion:**

These results underscore the critical role of multi-stakeholder interaction in advancing healthcare carbon neutrality. The study provides both theoretical insights and practical policy implications for designing more effective carbon neutrality strategies in the healthcare sector.

## 1 Introduction

In the 21st-century global agenda, climate change has emerged as an urgent problem with significant implications for human health (1) and wellbeing (2). As a key component of societal infrastructure, the healthcare industry's energy usage and greenhouse gas emissions during operation increasingly draw attention. Statistically speaking, the carbon emissions of the worldwide healthcare industry account for roughly 5% of the global total emissions (3), while certain industrialized nations, such as the United States, even exceed 10% (4). These data highlight the urgency and importance of reducing carbon emissions in the healthcare industry.

Against this backdrop, the healthcare industry has adopted the goal of medical carbon neutrality, aiming for net-zero carbon emissions by reducing emissions, improving efficiency, utilizing sustainable energy, and implementing carbon-offset technologies, thus balancing greenhouse gas outputs in clinical, operational, and supply chain processes with equivalent removals to achieve a net-zero environmental impact. For instance, the UK's National Health Service (NHS) commitment to carbon neutrality, targeting net-zero emissions by 2040 (5), exemplifies the emission reduction initiatives across the worldwide healthcare industry. The team from Solihull Hospital at the University Hospital of Birmingham in the UK completed the world's first carbon-neutral surgery in May 2022, reducing carbon emissions by 80% through equipment optimization, energy substitution, and other measures (6). The Cleveland Clinic in the United States has effectively decreased energy usage and carbon emissions via the adoption of energy-efficient practices and sustainable construction requirements. It aims to attain carbon neutrality by 2030 (7). Umea University Hospital in Sweden has lowered carbon emissions by 40 to 70 times relative to in-person treatment using remote rehabilitation therapy (8), this remarkable reduction exemplifies the pivotal role of digital therapeutics in advancing medical carbon neutrality.

The aforementioned instances illustrate that technology innovation and system optimization may proficiently regulate carbon emissions within the healthcare industry. Currently, the research focal points in healthcare carbon neutrality include energy management, the establishment of sustainable supply chains, the advancement and implementation of low-carbon medical technology, patient education (9), and public engagement. These studies focus on reducing the direct carbon emissions of the healthcare industry. Concurrently, these investigations aim to enhance the efficiency and quality of medical services while indirectly mitigating carbon emissions resulting from the increase in medical demand. In addition, the carbon neutrality measures in the healthcare industry also entail various levels, including policy formulation, financial investment, technological transfer, and public collaboration. Ultimately, these examples highlight that achieving medical carbon neutrality requires a comprehensive approach—one that combines stringent policy frameworks, strategic investment, cutting-edge technological innovations, and collaborative stakeholder engagement—to redefine healthcare as a sustainable, climate-resilient ecosystem.

The attainment of carbon neutrality in the medical industry may mitigate climate change, boost the efficiency of medical services, and bolster the resilience of the public health system on a worldwide scale. It is a crucial metric for advancing global sustainable development. Accordingly, we examined the policies and practices of different nations and regions in promoting medical carbon neutrality and developed a four-party evolutionary game model. This model examines the evolutionary dynamics among four principal roles: government, public hospitals, pharmaceutical enterprises, and the public. Subsequently, we delineate the strategic roles and decision-making environments of each participant inside the four-party game structure: the government acts as a worldwide policymaker and regulator, capable of directing the healthcare industry's low-carbon growth via law and financial incentives. Public hospitals, as direct partners in carbon neutrality initiatives, have simultaneous difficulties of improving healthcare service quality while minimizing environmental effect. Pharmaceutical enterprises serve as innovators and solution providers in this process. The public's health knowledge and consumption habits significantly influence medical carbon neutrality as end-users of healthcare services.

Furthermore, under uncertainty, decision-making processes are often delayed as stakeholders weigh long-term impacts (10). Therefore, incorporating time delay into the game framework is critical for accurately simulating strategic evolution and coordination among the four parties. This model contributes to a deeper understanding of how policy design and stakeholder behavior collectively shape the pathway toward achieving carbon-neutral healthcare systems.

## 2 Related literature

### 2.1 Medical carbon neutrality

The healthcare industry plays an important role in global carbon emissions and is currently facing significant sustainable development challenges. According to data from the World Health Organization (WHO), the global healthcare industry accounts for ~5% of total greenhouse gas emissions. If viewed as a country, it would become the fifth largest source of global emissions (11). The carbon emissions in the medical industry mainly come from energy consumption, anesthesia gases, the use of disposable products, and transportation and travel. Hospitals, especially operating rooms, have become the main source of carbon emissions due to their high energy consuming equipment (12, 13). Additionally, the use of anesthetics such as desflurane (14) and disposable medical devices such as sharps containers (15) greatly increases the carbon footprint. The transportation of patients and drugs (16) are also major contributors to carbon emissions in the healthcare industry. To address the above issues and achieve medical carbon neutrality, emission reduction strategies are essential. Currently, the main global emission reduction strategies include telemedicine (17), optimized management of medical equipment and operating room energy, and the use of low-carbon energy. Telemedicine, especially in replacing patients' short distance visits, has been proven to reduce carbon emissions by 40–70 times (16). By optimizing the design of operating rooms, using low global warming potential (GWP) anesthetics (18), and reusable medical devices (19), medical institutions can significantly reduce their carbon footprint. Furthermore, promoting clean energy (such as solar energy) and energy-saving technologies, as well as strengthening the classification and recycling management of medical waste, are also important paths for medical carbon neutrality (20). However, despite the increasing number of emission reduction measures and strategies for medical carbon neutrality, its implementation still faces many challenges. Firstly, the insufficient carbon emission data in the healthcare industry limits the comprehensive evaluation of emission reduction effects, especially the lack of sufficient empirical research on the comprehensive benefits of remote healthcare and low-carbon energy use throughout the entire lifecycle (8). Secondly, the lack of cross departmental cooperation and policy support makes it difficult to effectively implement carbon reduction measures throughout the entire healthcare system (21). The promotion of carbon neutrality in the healthcare industry requires not only technological innovation, but also policy incentives (21) and the transformation of industry culture (22). Strengthening cooperation between medical institutions, governments, patients, and suppliers, and promoting carbon footprint accounting and emission reporting systems in the healthcare industry will be key to achieving carbon neutrality in the future (21). In conclusion, attaining carbon neutrality in the healthcare industry demands both a reduction in energy consumption and an improvement in the efficiency of medical facilities and equipment, along with the promotion of multi-party collaboration through social engagement, policy suggestions, and technological progress. Despite facing certain challenges, the healthcare industry is expected to achieve carbon neutrality goals in the future by continuously improving technological means, strengthening policy implementation, and promoting public participation, making positive contributions to global environmental protection and public health.

### 2.2 The application of game theory in the research of medical carbon neutrality

The application of game theory in medical carbon neutralization has gradually attracted attention, which is mainly reflected in the strategic design of optimizing resource allocation, promoting cooperation and reducing carbon emissions. Recently, researchers have proposed several models based on game theory to meet the challenge of carbon neutrality in the medical field. Foremost, the game model of carbon trading market provides a theoretical framework for carbon emissions trading through blockchain Technology (23). Secondly, the cooperative game in medical waste treatment provides new ideas for reducing carbon emissions. Zhao et al. (24) constructed a tripartite game model among the government, medical institutions, and disposal enterprises, which promotes the resource utilization and disposal of medical waste through reasonable incentive and punishment mechanisms and reduces carbon emissions. The game model of the medical supply chain also demonstrates the potential of game theory in resource optimization. By mobilizing the game behavior of hospitals, patients, and the government, effective allocation of resources can be achieved, reducing the overall carbon emissions of the healthcare system (25). Yu (26) optimized internal control in public hospital supply chains through a game theory combination weighting method (AHP-entropy weighting method), established a comprehensive evaluation index system, and provided a new approach for the scientific management of medical supply chains. In addition, the dynamic regulatory and incentive model involving multiple parties also provides practical guidance for medical carbon neutrality (27). Although these game models provide theoretical basis for medical carbon neutrality, they still face challenges in data quantification, mechanism design, and cross domain integration. In the future, by combining evolutionary game theory with empirical data (13), more accurate carbon game frameworks will be developed, especially the application of technologies such as blockchain, which will provide more feasible solutions for medical carbon trading.

Evolutionary game theory is based on the assumption of bounded rationality, emphasizing the group dynamic evolution of strategies rather than individual optimal decisions, which is highly consistent with the complex interactions involving multiple entities (government, medical institutions, patients, suppliers, etc.) in medical carbon neutrality (28). Its core tools include Evolutionary Stability Strategy (ESS) (29), replicative dynamic (30), and reward and punishment mechanism design (31, 32). This provides a powerful tool for analyzing the long-term stability of various subject strategies in the context of medical carbon neutrality. In the study of specific application scenarios, the design of policy incentives is the primary field of evolutionary game theory. Scholars constructed a dynamic reward and punishment model including carbon tax, carbon subsidy and purchasing restriction to analyze the evolution path of healthcare organizations' strategies under different incentive strengths. For example, the National Health Service (NHS) in the UK requires providers to have a carbon reduction plan and imposes a purchasing ban on those who fail to meet the target (33, 34); the results of the game model show that when the penalty cost is higher than the abatement cost, low-carbon cooperation gradually becomes an evolutionarily stable strategy. Guo et al. (35) constructed a four-party evolutionary game model and pointed out that carbon prices, subsidy intensity, and punishment measures significantly affect the emission reduction decisions of supply chain enterprises. Among them, suppliers are sensitive to carbon prices, while manufacturers are more concerned about subsidies. This provides a reference for the government to assist in the implementation of medical carbon neutrality. Similarly, green technology promotion (e.g., introduction of solar energy facilities) can be evaluated through an evolutionary game framework to assess the impact of initial investment sharing vs. long-term energy returns on healthcare organizations' decision-making (36, 37). Evolutionary game theory also has forward-looking significance in patient participation and behavior guidance. Zhang et al. (38) used replicated dynamic equations to simulate patients' choices between remote healthcare and offline visits, and found that when carbon footprint information is transparent and the cost difference does not exceed 10%, the proportion of patients who prefer low-carbon diagnosis and treatment significantly increases. In addition, strategic synergy between physicians and patients through shared decision-making models can reduce carbon emissions by 20% to 30% in preventive healthcare promotion. This highlights the importance of public participation in building a carbon-neutral system for healthcare (39). However, although evolutionary game theory has made many advances in the field of healthcare carbon neutrality, scholars have mostly used the two-party or three-party evolutionary game approach to study the healthcare carbon neutrality problem, while the four-party evolutionary game approach to study healthcare carbon neutrality can further enrich the application of game theory in this field.

### 2.3 Application of time delays in supply chain carbon neutrality

Time delay, defined as the temporal delay between a system's input and its corresponding response, are commonly modeled by delay differential equations (DDEs). Such delays may manifest as discrete delay, distributed (continuous) delays, or impulsive delays (40). At present, time delays are widely employed across various disciplines. They occur in engineering systems (41), biological models (40), neural network architectures (42, 43), and in economic as well as traffic-flow analyses (44).

Time delays are also gradually receiving attention in the research of medical carbon neutrality technology. Biobased medical materials, such as surgical instrument packaging, can delay the release of CO_2_ beyond the target lifespan (such as 100 years) by extending their carbon storage time, and their emission reduction value is similar to permanent storage (45). From the perspective of technology promotion, the large-scale application of low-carbon drugs and devices that replace high GWP inhalers (such as salbutamol MDI) faces multiple delays in research and development verification, production transformation, and clinical acceptance, and usually takes several years to achieve significant emission reduction and efficiency improvement (46). Digitization and telemedicine are also expected to take 5–10 years to achieve significant results in patient transportation carbon emissions due to changes in population behavior habits and lagging infrastructure construction (47). At the policy and governance level, Portnoy et al. (48) draw on Norway's phased implementation of cervical cancer prevention policies to demonstrate how multi-stage evaluation processes can delay emissions reductions. Specifically, the approval and standard-setting for novel environmental technologies—such as low-carbon anesthesia protocols—are often prolonged by these successive assessments, thereby slowing the pace of decarbonization. Tennison et al. found that, between 1990 and 2019, the UK NHS achieved a 25% reduction in emissions by phasing out CFC-based inhalers, decarbonizing the electricity grid, and optimizing its supply chain; however, Scope 3 emissions (from pharmaceuticals and devices) remain difficult to curb due to delays in multinational production and procurement (49). Current literature reveals that research on time delays in medical supply chains and their carbon neutrality implications remains relatively scarce. Therefore, in this study, we integrated the time delay model into the four-party evolutionary game framework, enriching the research on time delay in the context of medical carbon neutrality.

Although some scholars have used game theory to study the issue of medical carbon neutrality, they still face challenges in mechanism design and cross disciplinary integration. Therefore, we use evolutionary game theory to study the issue of medical carbon neutrality and construct a more accurate framework for medical carbon neutrality games. Meanwhile, most existing literature has used two-party and tripartite evolutionary games to study medical carbon neutrality. Hence, we adopt the four-party evolutionary game method to investigate the issue of medical carbon neutrality. Finally, we incorporated time delays into the four-party evolutionary game model and further discussed the impact of time delays on medical carbon neutrality.

Table 1 summarizes the differences between our study and the existing literature reviewed previously. Specifically, most scholars use a tripartite evolutionary game model to study the carbon neutrality issue in the healthcare industry. Our research, however, has developed a four-party evolutionary game model that incorporates time delay factors, offering a new perspective for implementing carbon neutrality strategies in the medical industry. It further strengthens the literature on healthcare carbon neutrality by adjusting incentive and punishment policy mechanisms for stakeholders within a broader environmental sustainability framework.

**Table 1:** Comparison between our study and correlated literature.

**Correlative literature**	**Medical carbon neutrality**	**Game theory**	**Four-party evolutionary game**	**Time delays**
17	√			
20	√			
25		√		
26	√	√		
37		√	√	
50	√			√
Our study	√	√	√	√

## 3 Problem description and model assumptions

### 3.1 Problem description

The issues of energy consumption and carbon emissions in the healthcare industry have become a focus of social concerns. Certain pharmaceutical enterprises prioritize economic gains above their environmental obligations, leading to inadequate investment in emission reduction initiatives. The government, as policymakers and regulators, may direct and oversee public hospitals and pharmaceutical enterprises by establishing pertinent regulations to advance carbon neutrality in the healthcare industry. Public hospitals, as healthcare providers, ought to lead in sustainable healthcare practices by minimizing carbon emissions through enhanced energy management and service quality, while collaboratively assuming responsibility with governments to advance the green transformation of the healthcare industry. However, conflicts of interest between public hospitals and pharmaceutical enterprises, along with insufficient governmental oversight, have emerged as ambiguous elements in attaining medical carbon neutrality and protecting public health rights. The public, as the primary consumers of healthcare services, is essential in advancing the sustainable growth of the medical business. But the fact is that insufficient public understanding of and participation in green healthcare services has become a major barrier to achieving carbon neutrality in healthcare.

**Figure 1:**
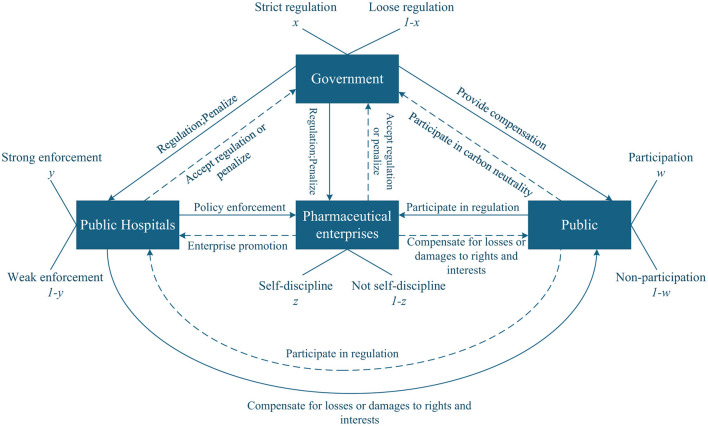
Relationship diagram of four players in the game of medical carbon neutrality.

Collaboration and games among the government, public hospitals, pharmaceutical enterprises, and the public are essential for achieving carbon neutrality in the healthcare industry. The interrelated strategies of participants create a dynamic environment where each player's choice affects and is affected by others. Figure 1 illustrates the logical links among the game elements of the four players.

### 3.2 Model assumption

#### 3.2.1 Hypothesis 1

Government, public hospitals, pharmaceutical enterprises, and the public are selected as the subjects of the four-party game. The probability of government enforcing strict regulation or loose regulation is (*x*, 1−*x*). The probability of public hospitals performing strong enforcement or weak enforcement is (*y*, 1−*y*). The probability of pharmaceutical enterprises adopting self-discipline or not self-discipline is (*z*, 1−*z*). The probability that the public chooses participation or non-participation is (*w*, 1−*w*). Here, *x*, *y*, *z*, *w*∈[ 0, 1].

#### 3.2.2 Hypothesis 2

We assume that the strict regulation cost of the government is *C*_*g*_. If the public hospitals carry out strong enforcement, pharmaceutical enterprises practice self-discipline, which can benefit the government, and we assume that this benefit is *R*_*g*_. Additionally, under public participation, this scenario can enhance the government's social credibility, represented by *k*_*g*_. Conversely, the government will incur losses *L*_*g*_ if either condition is met: (1) public hospitals adopt weak enforcement strategies, or (2) pharmaceutical enterprises engage in not self-discipline practices. For the public, the cost of participation is defined as *C*_*p*_. Meanwhile, when public hospitals choose strong enforcement and pharmaceutical enterprises choose self-discipline, the public will gain benefits *S*_*p*_. While when the above conditions are not met, the public will suffer losses *L*_*p*_.

#### 3.2.3 Hypothesis 3

Under strict government regulation, if public hospitals strongly enforce medical carbon neutrality policies and pharmaceutical enterprises adopt self-discipline strategies, they can receive government rewards, denoted as *R*_*h*_ and *R*_*c*_, respectively. Simultaneously, with public participation, if public hospitals strongly enforce medical carbon neutrality policies and pharmaceutical enterprises adopt self-discipline strategies, the credibility of both will be enhanced, denoted as *k*_*h*_ and *k*_*c*_, respectively. Assume the cost of strong enforcement for public hospitals is *C*_*h*_, and the cost of self-discipline for pharmaceutical enterprises is *C*_*c*_. If public hospitals choose weak enforcement, they can obtain short-term benefits *S*_*h*_. However, if the government imposes strict regulations at this time, the public hospitals will be required to pay a fine *D*_*h*_ to the government. Similarly, when pharmaceutical enterprises choose not self-discipline, they can obtain short-term income, which is *S*_*c*_. If the government carries out strict regulations in this scenario, pharmaceutical enterprises need to pay a fine *D*_*c*_ to the government.

#### 3.2.4 Hypothesis 4

The fines paid by public hospitals and pharmaceutical enterprises can serve as government revenue. Furthermore, when the public participates, these fines can be used to compensate the public. Suppose that α indicates the proportion of penalties obtained by the government during strict regulation, and 1−α expresses the proportion allocated to public compensation obtained by the public when the public participates, where α ∈ (0,1). It is essential that the public can only receive compensation if they participate when the government enforces strict supervision; otherwise, no compensation will be provided.

The definitions of the main parameter symbols are summarized in Table 2.

**Table 2:** Parameter definitions.

**Parameters**	**Definition**	**Parameters**	**Definition**
*R*_*g*_	The benefits the government can obtain when public hospitals strongly enforce medical carbon neutrality policies and pharmaceutical enterprises adopt self-discipline strategies.	*S*_*h*_	Short-term benefits of public hospitals under weak enforcement of medical carbon neutrality policies.
*R*_*h*_	Under strict government regulation and self-discipline of pharmaceutical enterprises, the benefits of public hospitals' strong enforcement of medical carbon neutrality policies	*S*_*c*_	Short-term benefits of pharmaceutical enterprises under not self-discipline strategies.
*R*_*c*_	Against the backdrop of strict government regulation and strong enforcement of medical carbon neutrality policies by public hospitals, pharmaceutical enterprises have gained benefits from self-discipline.	*D*_*h*_	Under strict government regulations, public hospitals are required to pay fines for weak enforcement of carbon neutrality strategies.
*k*_*g*_	The credibility enhanced by strict government regulation in the context of public participation.	*D*_*c*_	Under strict government regulations, pharmaceutical enterprises are required to pay fines for adopting not self-discipline strategies.
*k*_*h*_	The credibility enhanced by the strong enforcement of medical carbon neutrality policies by public hospitals in the context of public participation	*S*_*p*_	The benefits obtained by the public under the strong enforcement of medical carbon neutrality policies in public hospitals and the self-discipline of pharmaceutical enterprises
*k*_*c*_	The credibility enhanced by the self-discipline of pharmaceutical enterprises in the context of public participation.	*L*_*p*_	The public suffers losses when public hospitals adopt a weak enforcement strategy and pharmaceutical enterprises lack self-discipline.
*L*_*g*_	Losses borne by the government due to weak enforcement in public hospitals or not self-discipline in pharmaceutical enterprises	*C*_*p*_	The cost to the public when adopting a participation strategy
*x*	Probability of strict government regulation	*C*_*g*_	The cost for the government to adopt a strict regulation strategy
*y*	Probability of strong enforcement in public hospitals	*C*_*h*_	The cost of a strong enforcement strategy in public hospitals
*z*	Probability of self-discipline in pharmaceutical enterprises	*C*_*c*_	The cost of the self-discipline strategy in pharmaceutical enterprises
*w*	Probability of public participation	α	Under strict government regulations, the proportion of fines imposed on hospitals due to weak enforcement and not self-discipline by enterprises is attributed to the government

## 4 Stability analysis of players' strategy choices

Based on Hypotheses 1–4 in Section 3, a game payment matrix of the government, public hospitals, pharmaceutical enterprises, and the public are constructed under different strategy choices, which are shown in Table 3.

**Table 3:** Payoff matrix for the quadripartite game.

**Government**	**Public hospitals**	**Self-discipline of pharmaceutical enterprises z**		**Not self-discipline of pharmaceutical enterprises 1-z**	
		**Public participation w**	**Public non-participation 1-w**	**Public participation w**	**Public non-participation 1-w**
x (strict regulation)	y (strong enforcement)	*R*_*g*_−*C*_*g*_−*R*_*c*_−*R*_*h*_+*k*_*g*_	*R*_*g*_−*C*_*g*_−*R*_*h*_−*R*_*c*_	α*D*_*c*_−*C*_*g*_−*L*_*g*_	*D*_*c*_−*C*_*g*_−*L*_*g*_
		*R*_*h*_−*C*_*h*_+*k*_*h*_	*R*_*h*_−*C*_*h*_	−*C*_*h*_+*k*_*h*_	−*C*_*h*_
		*R*_*c*_−*C*_*c*_+*k*_*c*_	*R*_*c*_−*C*_*c*_	*S*_*c*_−*D*_*c*_−*k*_*c*_	*S*_*c*_−*D*_*c*_
		*S*_*p*_−*C*_*p*_	*S*_*p*_	(1−α)*D*_*c*_−*C*_*p*_−*L*_*p*_	−*L*_*p*_
	1-y (weak enforcement)	α*D*_*h*_−*C*_*g*_−*L*_*g*_	*D*_*h*_−*C*_*g*_−*L*_*g*_	α(*D*_*h*_+*D*_*c*_)−*C*_*g*_−*L*_*g*_	*D*_*h*_+*D*_*c*_−*C*_*g*_−*L*_*g*_
		*S*_*h*_−*D*_*h*_−*k*_*h*_	*S*_*h*_−*D*_*h*_	*S*_*h*_−*D*_*h*_−*k*_*h*_	*S*_*h*_−*D*_*h*_
		−*C*_*c*_+*kc*	−*C*_*c*_	*S*_*c*_−*D*_*c*_−*k*_*c*_	*S*_*c*_−*D*_*c*_
		(1−α)*D*_*h*_−*C*_*p*_−*L*_*p*_	−*L*_*p*_	(1−α)(*D*_*h*_+*D*_*c*_)−*C*_*p*_−*L*_*p*_	−*L*_*p*_
1-x (loose regulation)	y (strong enforcement)	*R*_*g*_−*k*_*g*_	*R*_*g*_	−*L*_*g*_−*k*_*g*_	−*L*_*g*_
		−*C*_*h*_+*k*_*h*_	−*C*_*h*_	−*C*_*h*_+*k*_*h*_	−*C*_*h*_
		−*C*_*c*_+*k*_*c*_	−*C*_*c*_	*S*_*c*_−*k*_*c*_	*S*_*c*_
		*S*_*p*_−*C*_*p*_	*S*_*p*_	−*C*_*p*_−*L*_*p*_	−*L*_*p*_
	1-y (weak enforcement)	−*L*_*g*_−*k*_*g*_	−*L*_*g*_	−*L*_*g*_−*k*_*g*_	−*L*_*g*_
		*S*_*h*_−*k*_*h*_	*S*_*h*_	*S*_*h*_−*k*_*h*_	*S*_*h*_
		−*C*_*c*_+*k*_*c*_	−*C*_*c*_	*S*_*c*_−*k*_*c*_	*S*_*c*_−*k*_*c*_
		−*L*_*p*_−*C*_*p*_	−*L*_*p*_	−*L*_*p*_−*C*_*p*_	−*L*_*p*_

### 4.1 Stability analysis for government

From the game payment matrix, it can be seen that the expected benefits for a government with strict regulation is *Ex*_1_, the expected benefits for a government with loose regulation is *Ex*_2_, and the average expected income for the government is $\bar{E}x$:


(1) $$\begin{array}{l} Ex_{1}=yzw\left(R_{g}-C_{g}-R_{h}-R_{c}+k_{g}\right)+yz\left(1-w\right)\\ \;\left(R_{g}-C_{g}-R_{h}-R_{c}\right)+y\left(1-z\right)w\left(\alpha D_{c}-C_{g}-L_{g}\right)\\ \;+y\left(1-z\right)\left(1-w\right)\left(D_{c}-C_{g}-L_{g}\right)+\left(1-y\right)\\ \;zw\left(\alpha D_{h}-C_{g}-L_{g}\right)+\left(1-y\right)z\left(1-w\right)\left(D_{h}-C_{g}-L_{g}\right)\\ \;+\left(1-y\right)\left(1-z\right)w\left[\alpha \left(D_{c}+D_{h}\right)-C_{g}-L_{g}\right]\\ \;+\left(1-y\right)\left(1-z\right)\left(1-w\right)\left(D_{h}+D_{c}-C_{g}-L_{g}\right)\; \end{array}$$

(2) $$\begin{array}{l} Ex_{2}=yzw\left(R_{g}-C_{g}\right)+yz\left(1-w\right)R_{g}+y\left(1-z\right)\\ \;w\left(-L_{g}-k_{g}\right)+y\left(1-z\right)\left(1-w\right)\left(-L_{g}\right)+\left(1-y\right)\\ \;zw\left(-L_{g}-k_{g}\right)+\left(1-y\right)z\left(1-w\right)\left(-L_{g}\right)+\left(1-y\right)\left(1-z\right)\\ \;w\left(-L_{g}-k_{g}\right)+\left(1-y\right)\left(1-z\right)\left(1-w\right)\left(-L_{g}\right)\; \end{array}$$

(3) $$\begin{array}{l} \bar{E}x=xEx_{1}+\left(1-x\right)Ex_{2}\; \end{array}$$

The replication of dynamic (50) is the core of evolutionary game theory, which describes the evolution process of the frequency of each player's strategy. In this paper, the replication dynamic equation of the government can help us better understand the impact of different strategies of the government as a subject on healthcare carbon neutrality. Based on this, we calculate the government's replication dynamic equation based on its expected and average benefits as follows:


(4) $$\begin{array}{c} F\left(x\right)=\frac{dx}{dt}=x\left(Ex_{1}-Ex_{2}\right)\\ =x\left(1-x\right)\left(Ex_{1}-\bar{E}x\right)\\ =x(1-x)\{wk_{g}-C_{g}-y[(\alpha w-w+1)D_{h}+z(R_{c}+R_{h}-wC_{g})]\\ -(w-\alpha w-1)(D_{c}+D_{h})-z[(\alpha -1)w+1]D_{c}\} \end{array}$$

The stability analysis of replicating dynamic equations is a touchstone for testing the feasibility of economic equilibrium evolution. This can provide dynamic optimization directions for institutional design. Therefore, we perform first-order differentiation on the government's replication dynamic equation for stability analysis. The first derivative of *F*(*x*) is as follows:


(5) $$\begin{array}{l} F^{\prime }(x)=(1-2x)\{wk_{g}-C_{g}-y[(\alpha w-w+1)D_{h}\\ \;+z(R_{c}+R_{h}-wC_{g})]-(w-\alpha -1)(D_{c}+D_{h})\\ \;-z[(\alpha -1)w+1]D_{c}\} \end{array}$$

It can be seen that whether the government chooses strict regulation depends on the cost and benefit of its different regulatory strategies and the probability of the other three parties' decision-making choices. According to the stability theorem of differential equations, the government's choice of strict supervision in a steady state must satisfy conditions *F*(*x*) = 0 and *F*′(*x*) < 0.

#### 4.1.1 Proposition 1

When *y*>*y*_1_, *z*>*z*_1_, *w*<*w*_1_, the government's stable strategy is loose regulation. When *y*<*y*_1_, *z*<*z*_1_, *w*>*w*_1_, the government's stable strategy is strict regulation. When *y* = *y*_1_, *z* = *z*_1_, *w* = *w*_1_, the government's stable strategy cannot be determined. The threshold value is as follows:


(6) $$\{\begin{array}{c} y_{1}=\frac{wk_{g}-C_{g}-(w-\alpha w-1)(D_{c}+D_{h})-z[(\alpha -1)w+1]D_{c}}{\left(\alpha w-w+1)D_{h}+z(R_{c}+R_{h}-wC_{g}\right)}\\ z_{1}=\frac{wk_{g}-C_{g}-y(\alpha w-w+1)D_{h}-(w-\alpha w-1)D_{c}}{y(R_{c}+R_{h}-wC_{g})+[(\alpha -1)w+1]D_{c}}\\ w_{1}=\frac{(z-1)D_{c}+(y-1)D_{h}+zy(R_{c}+R_{h})+C_{g}-wk_{g}}{y[(1-\alpha )D_{h}+zC_{g}]+(\alpha -1)(D_{c}+D_{h})-z(\alpha -1)D_{c}} \end{array}$$

Among them, *y*_1_ represents the threshold of public hospitals under the evolution of government strategies, which is the boundary point between weak or strong enforcement strategies adopted by public hospitals. *z*_1_ represents the threshold for pharmaceutical enterprises under the evolution of government strategies, which is the boundary between self-discipline or not self-discipline strategies adopted by pharmaceutical enterprises. *w*_1_ represents the threshold of the public under the evolution of government strategies, which is the dividing point between the public's adoption of participation or non-participation strategies. We have taken the threshold y1 of public hospitals as an example to explain how to calculate the threshold. The specific calculation process is as follows:

Construct function *G*(*y, z, w*) = *wk*_*g*_−*C*_*g*_−*y*[(α*w*−*w*+1)*D*_*h*_+*z*(*R*_*c*_+*R*_*h*_−*wC*_*g*_)]−(*w*−α*w*−1)(*D*_*c*_+*D*_*h*_)−*z*[(α−1)*w*+1]*D*_*c*_, as *G*(*y, z, w*) = 0, where *y* is an unknown variable and *z* and *w* are definite, solve for the threshold $y_{1}=\frac{wk_{g}-C_{g}-\left(w-\alpha w-1\right)\left(D_{c}+D_{h}\right)-z\left[\left(\alpha -1\right)w+1\right]D_{c}}{\left(\alpha w-w+1\right)D_{h}+z\left(R_{c}+R_{h}-wC_{g}\right)}$ of *y*. Similarly, the process of solving the threshold z1 for pharmaceutical companies and the threshold w1 for the public is also the same.

#### 4.1.2 Proof of proposition 1

As can be seen from the previous text, *G*(*y, z, w*) = *wk*_*g*_−*C*_*g*_−*y*[(α*w*−*w*+1)*D*_*h*_+*z*(*R*_*c*_+*R*_*h*_−*wC*_*g*_)]−(*w*−α*w*−1)(*D*_*c*_+*D*_*h*_)−*z*[(α−1)*w*+1]*D*_*c*_, as $\frac{\partial G\left(y,z,w\right)}{\partial y}<0$, *G*(*y, z, w*) is strictly decreasing function in terms of *y*. When *y*>*y*_1_, *G*(*y, z, w*) < 0, *F*(*x*)|_*x* = 0_ = 0 and *F*^′^(*x*)|*x* = 0 < 0, the equilibrium point *x* = 0 is asymptotically stable; when *y*<*y*_1_, *G*(*y, z, w*)>0,*F*(*x*)|_*x* = 1_ = 0 and *F*^′^(*x*)|*x* = 0 < 1, then *x* = 1 is asymptotically stable; when *y* = *y*_1_, *F*(*x*) = 0 and *F*′(*x*) = 0, the governments stabilization strategy cannot be determined. By condition $\frac{\partial G\left(y,z,w\right)}{\partial z}<0$ and $\frac{\partial G\left(y,z,w\right)}{\partial w}>0$, the influence of the critical thresholds *z*_1_ and *w*_1_ on the strategy's stability can be similarly demonstrated. The proof of Proposition 1 is complete.

Proposition 1 shows that in the process of medical carbon neutral policy enforcement, if the probability of strong enforcement of public hospitals and the probability of self-discipline of pharmaceutical enterprises increase, and the probability of public participation decreases, the government will choose the loose regulation strategy; on the contrary, the government will shift from loose regulation to strict regulation.

**Figure 2:**
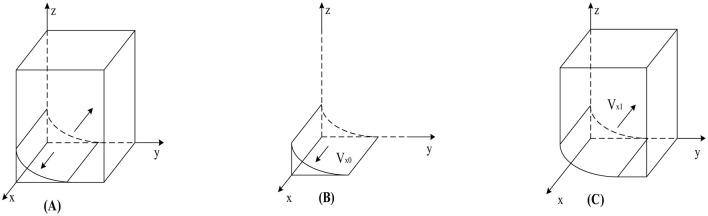
Phase diagram of government strategy selection. **(A)** *y* = *y*_1_. **(B)** *y* < *y*_1_. **(C)** *y* > *y*_1_.

According to Proposition 1, the phase diagram of government regulation strategy selection is drawn in Figure 2.

As shown in Figure 2, the volumes of regions *V*_*x*0_ and *V*_*x*0_ represent the probabilities of strict and loose government regulation, respectively. Since we cannot determine the specific values of the points on the y-axis in Figure 2, we set *z* = 0 and *w* = 0; simultaneously calculate $y=\frac{D_{c}-yD_{h}-C_{g}}{y\left(R_{c}+R_{h}\right)+D_{c}}$ based on *y*_1_. Finally, to better calculate the volumes of *V*_*x*0_ and *V*_*x*0_, we construct the equation $v=\frac{D_{c}-yD_{h}-C_{g}}{y\left(R_{c}+R_{h}\right)+D_{c}}$, the calculation yields are as follows:


(7) Vx0=01z0vd1ydx       =wkg−Cg−(w−αw−1)(Dc+Dh)Rc+Rh−Cg        ln[1+v(Rc+Rh−wCg)(αw−w+1)Dh]−[(α−1)w+1]Dc        ln{v(Rc+Rh−wCg)−(αw−w+1)Dh        ln[1+v(Rc+Rh−wCg)(αw−w+1)Dh]} 

(8) Vx1=1−01zv1d1ydx        =1−wkg−Cg−(w−αw−1)(Dc+Dh)Rc+Rh−Cg        ln[1+v(Rc+Rh−wCg)(αw−w+1)Dh]+[(α−1)w+1]Dc        ln{v(Rc+Rh−wCg)−(αw−w+1)Dh        ln[1+v(Rc+Rh−wCg)(αw−w+1)Dh]} 

#### 4.1.3 Corollary 1

Government choices regarding strict regulation strategies are affected by a variety of factors. These factors include the improvement of government credibility; the increase in fines paid by public hospitals adopting weak enforcement strategies; the increase in penalties paid by pharmaceutical enterprises adopting not self-discipline strategies; the reduction in government regulatory costs; and the reduction in the reward scale when public hospitals adopt strong enforcement strategies and pharmaceutical enterprises maintain self-discipline. If these conditions are met, the likelihood of the government implementing strict regulation will significantly increase. Conversely, governments tend to prefer loose regulation strategies if these conditions are reversed.

#### 4.1.4 Proof of corollary 1

By solving the first-order partial derivatives of the government's choice of the strict regulation probability *V*_*x*0_, with respect to *k*_*g*_, *D*_*c*_, *D*_*h*_, *C*_*g*_, *R*_*c*_, and *R*_*h*_, we can obtain: $\frac{\partial V_{x0}}{\partial k_{g}}>0$, $\frac{\partial V_{x0}}{\partial D_{c}}>0$, $\frac{\partial V_{x0}}{\partial D_{h}}>0$, $\frac{\partial V_{x0}}{\partial C_{g}}<0$, $\frac{\partial V_{x0}}{\partial R_{c}}<0$,$\frac{\partial V_{x0}}{\partial R_{h}}<0$, certificate completed.

### 4.2 Stability analysis for public hospitals

From the game payment matrix, it can be seen that the expected benefits for the public hospitals with strong enforcement is *Ey*_1_, the expected benefits for the public hospital with weak enforcement is *Ey*_2_, and the average expected income for the public hospitals is $\bar{E}y$:


(9) $$\begin{array}{l} Ey_{1}=xzw\left(R_{h}-C_{h}+k_{h}\right)+xz\left(1-w\right)\left(R_{h}-C_{h}\right)+x\left(1-z\right)\\ \;w\left(-C_{h}+k_{h}\right)+x\left(1-z\right)\left(1-w\right)\left(-C_{h}\right)+\left(1-x\right)\\ \;zw\left(-C_{h}+k_{h}\right)+\left(1-x\right)z\left(1-w\right)\left(-C_{h}\right)+\left(1-x\right)\\ \;\left(1-z\right)w\left(-C_{h}+k_{h}\right)+\left(1-x\right)\left(1-z\right)\left(1-w\right)\left(-C_{h}\right)\; \end{array}$$

(10) $$\begin{array}{l} Ey_{2}=xzw\left(S_{h}-D_{h}-k_{h}\right)+xz\left(1-w\right)\left(S_{h}-D_{h}\right)+x\left(1-z\right)\\ \;w\left(S_{h}-D_{h}-k_{h}\right)+x\left(1-z\right)\left(1-w\right)\left(S_{h}-D_{h}\right)\\ \;+\left(1-x\right)zw\left(S_{h}-k_{h}\right)+\left(1-x\right)z\left(1-w\right)S_{h}+\left(1-x\right)\left(1-z\right)\\ \;w\left(S_{h}-k_{h}\right)+\left(1-x\right)\left(1-z\right)\left(1-w\right)S_{h}\; \end{array}$$

(11) $$\begin{array}{l} \bar{E}y=yEy_{1}+\left(1-y\right)Ey_{2}\; \end{array}$$

The replication dynamic equation of public hospitals can help us better understand the impact of different strategies of public hospitals on healthcare carbon neutrality. Based on this, we calculated the public hospitals' replication dynamic equation based on the expectations and average benefits of public hospitals. The equation for the replication dynamics of public hospitals is as follows:


(12) $$\begin{array}{l} F\left(y\right)=\frac{dy}{dt}=y\left(Ey_{1}-Ey_{2}\right)\\ \;=y\left(1-y\right)\left(Ey_{1}-\bar{E}y\right)\\ \;=y\left(1-y\right)\left[x\left(D_{h}+zR_{h}\right)+2wk_{h}-S_{h}-C_{h}\right]\; \end{array}$$

To conduct stability analysis on the replication dynamic equation of public hospitals and identify the impact of the other three parties on public hospital strategies, we performed first-order differentiation on the replication dynamic equation of public hospitals. The first derivative of *F*(*y*) is as follows:


(13) $$\begin{array}{l} F^{\prime }\left(y\right)=\left(1-2y\right)\left[x\left(D_{h}+zR_{h}\right)+2wk_{h}-S_{h}-C_{h}\right]\; \end{array}$$

From Equations 12, 13, it can be seen that the main factors affecting the strategy selection of public hospitals include when public hospitals enforce strong enforcement strategy, the government's strict regulation brings rewards *R*_*h*_ to public hospitals; the cost *C*_*h*_ of public hospitals adopting a strong enforcement strategy; the fine *D*_*h*_ that public hospitals have to pay when adopting a weak enforcement strategy under strict government regulation; the reputation *k*_*h*_ of public hospitals; and the short-term benefits *S*_*h*_ of public hospitals adopting a weak enforcement strategy. According to the stability theorem of differential equations, the steady-state conditions for selecting strong enforcement in public hospitals should be satisfied: *F*(*y*) = 0 and *F*′(*y*) < 0.

#### 4.2.1 Proposition 2

When *x*<*x*_1_, *z*<*z*_2_, *w*<*w*_2_, the stable strategy of public hospitals is to choose weak enforcement; When *x*>*x*_1_, *z*>*z*_2_, *w*>*w*_2_, the stable strategy of public hospitals is to choose strong enforcement; When *x* = *x*_1_, *z* = *z*_2_, *w* = *w*_2_, the public hospitals' stable strategy cannot be determined. The threshold value is as follows:


(14) $$\{\begin{array}{c} x_{1}=\frac{S_{h}+C_{h}-2wk_{h}}{D_{h}+zR_{h}}\\ z_{2}=\frac{S_{h}+C_{h}-xD_{h}-2wk_{h}}{xR_{h}}\\ w_{2}=\frac{S_{h}+C_{h}-xD_{h}-xzR_{h}}{2k_{h}} \end{array}$$

Among them, *x*_1_ represents the threshold of the government's strategy evolution in public hospitals, which is the boundary point between the government's adoption of strict regulatory strategies or loose regulatory strategies. *z*_2_ represents the threshold for pharmaceutical enterprises in the evolution of public hospital strategies, which is the boundary between the adoption of self-discipline strategies or non-self-discipline strategies by pharmaceutical enterprises. *w*_2_ represents the threshold of the public's participation in the evolution of public hospital strategies, which is the dividing point between the public's adoption of participation strategies or non-participation strategies. The calculation process has been explained in Section 4.1, so it will not be elaborated here.

#### 4.2.2 Proof of proposition 2

Construct the function *H*(*x, z, w*) = *x*(*D*_*h*_+*zR*_*h*_)+2*wk*_*h*_−*S*_*h*_−*C*_*h*_. Since $\frac{\partial H\left(x,z,w\right)}{\partial x}>0$, *H*(*x, z, w*) is an increasing function with regard to *x*. When *x*<*x*_1_, we have that *H*(*x, z, w*) < 0, *F*(*y*)|_*y* = 0_ = 0, *F*^′^(*y*)|*y* = 0 < 0, and then *y* = 0 has stability. When *x*>*x*_1_, *H*(*x, z, w*)>0, *F*(*y*)|_*y* = 1_ = 0, *F*^′^(*y*)|*y* = 0 < 0, and then *y* = 1 has stability. When *x* = *x*_1_, we can obtain that *H*(*x, z, w*) = 0, and then *F*(*y*) = 0 and *F*′(*y*) = 0,. Now, no stable strategy can be identified. By condition $\frac{\partial H\left(x,z,w\right)}{\partial z}<0$ and $\frac{\partial H\left(x,z,w\right)}{\partial w}<0$, the influence of the critical thresholds *z*_2_ and *w*_2_ on the strategy's stability can be similarly demonstrated. The proof of Proposition 2 is complete.

Proposition 2 indicates that during the enforcement of the medical carbon neutrality strategy, if the government chooses strict regulation, pharmaceutical enterprises choose self-discipline, and the probability of public participation increases, the probability of public hospitals choosing strong enforcement will also increase. Conversely, public hospitals will shift from strong enforcement to weak enforcement.

**Figure 3:**
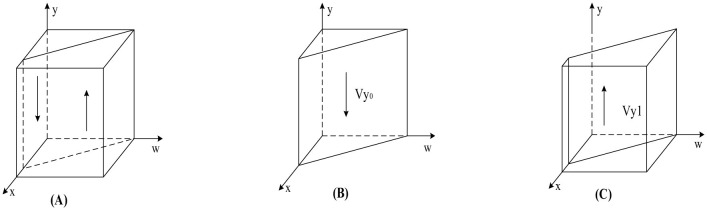
Phase diagram of public hospitals' strategy selection. **(A)** *x* = *x*_1_. **(B)** *x* < *x*_1_. **(C)** *x* > *x*_1_.

According to Proposition 2, Figure 3 shows the phase diagram for the strategy selection of public hospitals.

As can be seen from Figure 3, the volumes of the *V*_*y*0_ and *V*_*y*1_ sections represent the probability of weak and strong enforcement by public hospitals, respectively, which are calculated as follows:


(15) Vy0=01x01d1ydw =Sh+Ch−khDh+zRh

(16) Vy1=1−01x01d1ydw=1−Sh+Ch−khDh+zRh

#### 4.2.3 Corollary 2

Many factors influence public hospitals to adopt weak enforcement strategies. The factors include the increase in short-term incomes of public hospitals; public hospitals' costs increase when they adopt a strong enforcement strategy; the credibility of public hospitals declines; the reward from the government to the public hospitals is reduced when the public hospitals adopt a strong enforcement strategy; the government reduces penalties for public hospitals implementing weak enforcement strategies. If these conditions are met, the likelihood of the public hospitals implementing weak enforcement will significantly increase. Conversely, when these conditions reverse, public hospitals tend to shift toward strong enforcement strategies. The proof process is similar to the analysis of government strategy stability.

### 4.3 Stability analysis for pharmaceutical enterprises

From the game payment matrix, it can be seen that the expected benefits for the pharmaceutical enterprises with self-discipline is *Ez*_1_, the expected benefits for the pharmaceutical enterprises with not self-discipline is *Ez*_2_, and the average expected income for the pharmaceutical enterprises is $\bar{E}z$:


(17) $$\begin{array}{l} Ez_{1}=xyw\left(R_{c}-C_{c}+k_{c}\right)+xy\left(1-w\right)\left(R_{c}-C_{c}\right)+x\left(1-y\right)\\ \;w\left(-C_{c}+k_{c}\right)+x\left(1-y\right)\left(1-w\right)\left(-C_{c}\right)+\left(1-x\right)\\ \;yw\left(-C_{c}+k_{c}\right)+\left(1-x\right)y\left(1-w\right)\left(-C_{c}\right)+\left(1-x\right)\left(1-y\right)\\ \;w\left(-C_{c}+k_{c}\right)+\left(1-x\right)\left(1-y\right)\left(1-w\right)\left(-C_{c}\right)\; \end{array}$$

(18) $$\begin{array}{l} Ez_{2}=xyw\left(S_{c}-D_{c}-k_{c}\right)+xy\left(1-w\right)\left(S_{c}-D_{c}\right)\\ \;+x\left(1-y\right)w\left(S_{c}-D_{c}-k_{c}\right)\\ \;+x\left(1-y\right)\left(1-w\right)\left(S_{c}-D_{c}\right)\\ \;+\left(1-x\right)yw\left(S_{c}-k_{c}\right)+\left(1-x\right)y\left(1-w\right)S_{c}\\ \;+\left(1-x\right)\left(1-y\right)w\left(S_{c}-k_{c}\right)+\left(1-x\right)\left(1-y\right)\left(1-w\right)S_{c}\; \end{array}$$

(19) $$\begin{array}{l} \bar{E}z=zEz_{1}+\left(1-z\right)Ez_{2}\; \end{array}$$

The replication dynamic equation of pharmaceutical enterprises can help us better understand the impact of different strategies of pharmaceutical enterprises on healthcare carbon neutrality. Based on this, we calculated the replication dynamic equation for pharmaceutical enterprises based on their expectations and average benefits. The replication dynamic equation for pharmaceutical enterprises is as follows:


(20) $$\begin{array}{l} F\left(z\right)=\frac{dz}{dt}=z\left(Ez_{1}-\bar{E}z\right)=z(1-z)[x\left(D_{c}+yR_{c}\right)\\ \;+2wk_{c}-S_{c}-C_{c}]\; \end{array}$$

To conduct stability analysis on the replication dynamic equation of pharmaceutical enterprises and determine the impact of the other three parties on the strategy of pharmaceutical enterprises, we performed first-order differentiation on the replication dynamic equation of pharmaceutical enterprises. The first derivative of *F*(*z*) is as follows:


(21) $$\begin{array}{l} F^{\prime }\left(z\right)=\left(1-2z\right)\left[x\left(D_{c}+yR_{c}\right)+2wk_{c}-S_{c}-C_{c}\right]\; \end{array}$$

It can be seen from Equations 20, 21 that the main factors influencing the strategic choices of pharmaceutical enterprises include: under strict government regulation, the government rewards self-disciplined pharmaceutical enterprises with *R*_*c*_; the cost of self-discipline *C*_*c*_ for pharmaceutical enterprises; the reputation enhancement degree *k*_*c*_; the short-term benefits *S*_*c*_ from not self-discipline; and when the government strictly regulates, the pharmaceutical enterprises do not self-discipline, pay the fine *D*_*c*_. According to the stability theorem of differential equations, the steady-state conditions for selecting self-discipline in pharmaceutical enterprises should be satisfied: *F*(*z*) = 0 and *F*′(*z*) < 0.

#### 4.3.1 Proposition 3

When *x*<*x*_2_, *y*<*y*_2_, *w*<*w*_3_, the pharmaceutical enterprises' stable strategy is not self-discipline. When *x*>*x*_2_, *y*>*y*_2_, *w*>*w*_3_, the pharmaceutical enterprises' stable strategy is self-discipline; When *x* = *x*_2_, *y* = *y*_2_, *w* = *w*_3_, the pharmaceutical enterprises' stability strategy cannot be determined. The threshold value is as follows:


(22) $$\{\begin{array}{c} x_{1}=\frac{S_{c}+C_{c}-2wk_{c}}{D_{c}+zR_{c}}\\ z_{2}=\frac{S_{c}+C_{c}-xD_{c}-2wk_{c}}{xR_{c}}\\ w_{2}=\frac{S_{c}+C_{c}-xD_{c}-xzR_{c}}{2k_{c}} \end{array}$$

Among them, *x*_2_ represents the threshold of the government's strategic evolution in the pharmaceutical enterprises, which is the boundary point between the government's adoption of strict regulatory strategies or loose regulatory strategies. *y*_2_ represents the threshold of public hospitals under the evolution of pharmaceutical enterprises strategies, which is the boundary point for public hospitals to adopt strong enforcement strategies or weak enforcement strategies are implemented. *w*_3_ represents the threshold of the public in the evolution of pharmaceutical enterprises strategies, which is the dividing point between the public adopting participation strategies or not participating strategies. The specific calculation process has been explained in Section 4.1.

#### 4.3.2 Proof of proposition 3

Construct the function *P*(*x, y, w*) = *x*(*D*_*c*_+*yR*_*c*_)+2*wk*_*c*_−*S*_*c*_−*C*_*c*_. Because $\frac{\partial P\left(x,y,w\right)}{\partial x}>0$, *P*(*x, y, w*) is an increasing function with regard to *x*. When *x*<*x*_2_, we have that *P*(*x, y, w*) < 0, *F*(*z*)|_*z* = 0_ = 0, *F*^′^(*z*)|*z* = 0 < 0, and then *z* = 0 has stability. When *x*>*x*_2_, *P*(*x, y, w*)>0, *F*(*z*)|_*z* = 1_ = 0, *F*^′^(*z*)|*z* = 1 < 0, and then *z* = 1 has stability. When *x* = *x*_2_, we can obtain that *P*(*x, y, w*) = 0, and then *F*(*z*) = 0 and *F*′(*z*) = 0,. Now, no stable strategy can be identified. By condition $\frac{\partial P\left(x,y,w\right)}{\partial y}<0$ and $\frac{\partial P\left(x,y,w\right)}{\partial w}<0$, the influence of the critical thresholds *y*_2_ and *w*_3_ on the strategy's stability can be similarly demonstrated. The proof of Proposition 3 is complete.

Proposition 3 implies that during the implementation of medical carbon neutrality strategies, when the government enforces strict regulation, public hospitals demonstrate strong enforcement, and the probability of public participation increases, the probability of pharmaceutical enterprises practicing self-discipline also rises; Conversely, pharmaceutical enterprises will shift from self-discipline to not self-discipline.

**Figure 4:**
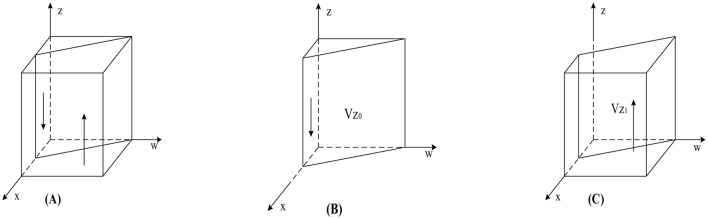
Phase diagram of pharmaceutical enterprises' strategy selection. **(A)** *w* = *w*_3_. **(B)** *w* < *w*_3_ **(C)** *w* > *w*_3_.

According to Proposition 3, the phase diagram for the strategy selection of a pharmaceutical enterprise is drawn, as shown in Figure 4.

As shown in Figure 4, the volumes corresponding to regions *V*_*z*0_ and *V*_*z*1_ represent the probabilities of pharmaceutical enterprises' not self-discipline and self-discipline, respectively. The calculation yields:


(23) Vz0=01w01d3xdz=Sc+Cc−kcDc+ykc

(24) Vz1=1−01w01d3xdz=1−Sc+Cc−kcDc+ykc

#### 4.3.3 Corollary 3

Pharmaceutical enterprises' choices regarding self-discipline strategies are affected by various factors. These factors include the increase of short-term profits of pharmaceutical enterprises; increased cost of self-discipline in pharmaceutical enterprises; when the government strictly regulates, the fines that pharmaceutical enterprises have to pay for no self-discipline are reduced; pharmaceutical enterprises' credibility declines. If these conditions are met, the probability of the pharmaceutical enterprises adopting not self-discipline will significantly increase. Conversely, if these conditions reverse, pharmaceutical enterprises tend to prefer self-discipline strategies.

### 4.4 Stability analysis for public

From the game payment matrix, it can be seen that the expected benefits for the public with participation is *Ew*_1_, the expected benefits for the public with non-participation is *Ew*_2_, and the average expected income for the public is $\bar{E}w$:


(25) $$\begin{array}{l} Ew_{1}=xyz\left(S_{p}-C_{p}\right)+xy\left(1-z\right)\left[\left(1-\alpha \right)D_{c}-C_{p}-L_{p}\right]\\ \;+x\left(1-y\right)z\left[\left(1-\alpha \right)D_{h}-C_{p}-L_{p}\right]+x\left(1-y\right)\left(1-z\right)\\ \;\left[\left(1-\alpha \right)\left(D_{h}+D_{c}\right)-L_{p}-C_{p}\right]+\left(1-x\right)yz\left(S_{p}-C_{p}\right)\\ \;+\left(1-x\right)y\left(1-z\right)\left(-C_{p}-L_{p}\right)+\left(1-x\right)\left(1-y\right)z\left(-L_{p}-C_{p}\right)\\ \;+\left(1-x\right)\left(1-y\right)\left(1-z\right)\left(-L_{p}-C_{p}\right) \end{array}$$

(26) $$\begin{array}{l} Ew_{2}=xyzS_{p}+xy\left(1-z\right)\left(-L_{p}\right)\\ \;+x\left(1-y\right)z\left(-L_{p}\right)+x\left(1-y\right)\left(1-z\right)\left(-L_{p}\right)\\ \;+\left(1-x\right)yzS_{p}+\left(1-x\right)y\left(1-z\right)\left(-L_{p}\right)+\left(1-x\right)\\ \;\left(1-y\right)z\left(-L_{p}\right)+\left(1-x\right)\left(1-y\right)\left(1-z\right)\left(-L_{p}\right)\; \end{array}$$

(27) $$\begin{array}{l} \bar{E}w=wEw_{1}+\left(1-w\right)Ew_{2}\; \end{array}$$

The public's replication of dynamic equations can help us better understand the impact of different public strategies on healthcare carbon neutrality. Based on this, we calculated the public's replication dynamics equation based on public expectations and average returns. The replication dynamic equation for pharmaceutical companies is as follows:


(28) $$\begin{array}{l} F\left(w\right)=\frac{dw}{dt}=w\left(Ew_{1}-\bar{E}w\right)=w(1-w)\{x(1-\alpha )(D_{c}+D_{h})\\ \;-x[(1-\alpha )yD_{h}+(1-\alpha )zD_{c}]-C_{p}\} \end{array}$$

To conduct stability analysis on the public replication dynamic equation and determine the impact of the other three parties on public strategy, we performed first-order differentiation on the public replication dynamic equation. The first derivative of *F*(*w*) is as follows:


(29) $$\begin{array}{l} F^{\prime }(w)=(1-2w)\{x(1-\alpha )(D_{c}+D_{h})\\ \;-x[(1-\alpha )yD_{h}+(1-\alpha )zD_{c}]-C_{p}\} \end{array}$$

It can be seen from Equations 28, 29 that the main factors affecting the public's strategy choice include the fine *D*_*h*_ that public hospitals have to pay when they adopt a weak enforcement strategy under strict government regulation, the fine *D*_*c*_ that pharmaceutical enterprises have to pay when they adopt a not self-discipline strategy, and the cost *C*_*p*_ when the public participates.

#### 4.4.1 Proposition 4

When *x*<*x*_3_, *y*>*y*_3_, *z*>*z*_3_, the public's stable strategy is non-participation; When *x*>*x*_3_, *y*<*y*_3_, *z*<*z*_3_, the public's stable strategy is participation; When *x* = *x*_3_, *y* = *y*_3_, *z* = *z*_3_, the public stability strategy cannot be determined. The threshold value is:


(30) $$\{\begin{array}{c} x_{3}=\frac{C_{p}}{(1-\alpha )(D_{c}+D_{h})-(1-\alpha )yD_{h}-(1-a)zD_{c}}\\ y_{3}=\frac{x(1-\alpha )(D_{c}+D_{h})-xz(1-\alpha )D_{c}-C_{p}}{x(1-\alpha )D_{h}}\\ z_{3}=\frac{x(1-\alpha )(D_{c}+D_{h})-xy(1-\alpha )D_{h}}{x(1-\alpha )D_{h}} \end{array}$$

Among them, *x*_3_ represents the threshold of the government under the evolution of public strategies, which is the boundary point between the government adopting strict regulatory strategies or loose regulatory strategies. *y*_3_ represents the threshold of public hospitals under the evolution of public strategies, which is the boundary point for public hospitals to adopt strong or weak enforcement strategies. *z*_3_ represents the threshold for pharmaceutical enterprises to adopt self-discipline strategies or not self-discipline strategies under the evolution of public policies. The specific calculation process has been explained in Section 4.1 and will not be further elaborated here.

#### 4.4.2 Proof of proposition 4

Construct the function *Q*(*x, y, z*) = *x*(1−α)(*D*_*c*_+*D*_*h*_)−*x*[(1−α)*yD*_*h*_+(1−α)*zD*_*c*_]−*C*_*p*_. Since $\frac{\partial Q\left(x,y,z\right)}{\partial x}>0$, *Q*(*x, y, z*) is an increasing function about *x*. When *x*<*x*_3_, we have that *Q*(*x, y, z*) < 0, *F*(*w*)|_*w* = 0_ = 0, *F*^′^(*w*)|*w* = 0 < 0, and then *w* = 0 has stability. When *x*>*x*_3_, *Q*(*x, y, z*)>0, *F*(*w*)|_*w* = 1_ = 0, *F*^′^(*w*)|*w* = 1 < 0, and then *w* = 1 has stability. When *x* = *x*_3_, we can obtain that *Q*(*x, y, z*) = 0, and then *F*(*w*) = 0 and *F*′(*w*) = 0,. Now, no stable strategy can be identified. By condition $\frac{\partial Q\left(x,y,z\right)}{\partial y}>0$ and $\frac{\partial Q\left(x,y,z\right)}{\partial w}>0$, the influence of the critical thresholds *y*_3_ and *z*_3_ on the strategy's stability can be similarly demonstrated. The proof of Proposition 4 is complete.

Proposition 4 suggests that during the implementation of medical carbon neutrality strategies, if the government adopts loose regulation, public hospitals adopt strong enforcement, and the probability of pharmaceutical enterprises adopting self-discipline increases, the public will opt for non-participation; Conversely, the public will shift from non-participation to participation.

**Figure 5:**
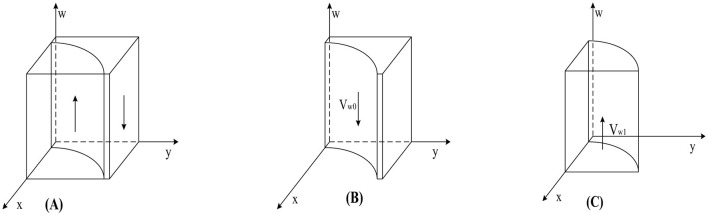
Phase diagram of public strategy selection. **(A)** *x* = *x*_3_. **(B)** *x* < *x*_3_. **(C)** *x* > *x*_3_.

According to Proposition 4, the phase diagram for the public's strategy selection is drawn, as shown in Figure 5.

As shown in Figure 5, the volumes corresponding to regions *V*_*w*0_ and *V*_*w*1_ represent the probabilities of public' participation and non-participation, respectively. The calculation yields:


(31) Vw0=01x01d3ydw=−Cp(1−α)Dhln[(1−α)(Dc+Dh)         −(1−α)yDh−(1−α)zDc] 

(32) Vw1=1−01d01ydw=1+Cp(1−α)Dhln[(1−α)(Dc+Dh)        −(1−α)yDh−(1− α)zDc]

#### 4.4.3 Corollary 4

When the public adopts participation strategies, the increased fines imposed on public hospitals for choosing weak enforcement strategies, the increased fines imposed on pharmaceutical enterprises for choosing not self-discipline strategies, and the reduction in public participation costs will all increase the probability of the public choosing participation strategies. Conversely, the public will opt for non-participation strategies. The proof process is similar to the stability analysis of government strategies.

## 5 Stability analysis of strategy combinations

In a four-party evolutionary game replication dynamic system, there are 16 potential strategy combination scenarios, each of which may become the equilibrium point of the group evolutionary game—conducting stability analysis on the equilibrium points of the replication dynamic system, which helps to grasp the emergence of different evolutionary scenarios. Furthermore, adjusting key parameters promotes the evolution of the game system toward Pareto optimal scenarios and avoids the emergence of poor game equilibria. The stability of the game players' strategy combination can be judged according to Lyapunov's first rule (51). If all eigenvalues of the Jacobian matrix (52) have negative genuine parts, the equilibrium point is an asymptotically evolutionarily stable strategy (ESS) (53). The equilibrium point is unstable if at least one eigenvalue of the Jacobian matrix has a positive real part. If all eigenvalues of the Jacobian matrix, except for zero eigenvalues, have negative real parts, the equilibrium point is in a critical state with uncertain stability. Based on the replication dynamics equations of each game player, the Jacobian matrix of the replication dynamic system is:


(33) $$\begin{array}{l} J=\left[\begin{array}{cccc} \partial F\left(x\right)/\partial x & \partial F\left(x\right)/\partial y & \partial F\left(x\right)/\partial z & \partial F\left(x\right)\partial w\\ \partial F\left(y\right)/\partial x & \partial F\left(y\right)/\partial y & \partial F\left(y\right)/\partial z & \partial F\left(y\right)/\partial w\\ \partial F\left(z\right)/\partial x & \partial F\left(z\right)/\partial y & \partial F\left(z\right)/\partial z & \partial F\left(z\right)/\partial w\\ \partial F\left(w\right)/\partial x & \partial F\left(w\right)/\partial y & \partial F\left(w\right)/\partial z & \partial F\left(w\right)/\partial w \end{array}\right]\; \end{array}$$

As a supervisory body, the government is responsible and obligated to implementing medical carbon neutrality strategies. To study the impact of government regulation strategies on system evolution through comparative analysis, we divided the 16 pure strategy equilibrium points into two scenarios: loose regulation and strict regulation. This can focus on the evolution and stability of public hospitals and pharmaceutical enterprises under regulatory strategies and provide a theoretical basis for building a more effective government regulatory system.

### 5.1 Asymptotic stability analysis of equilibrium points in replication dynamic systems under loose government regulation

During the implementation of medical carbon neutrality strategies, when the government's stable strategy is loose regulation, that is, when the following condition is satisfied: *wk*_*g*_−*C*_*g*_−*y*[(α*w*−*w*+1)*D*_*h*_+*z*(*R*_*c*_+*R*_*h*_−*wC*_*g*_)]−(*w*−α*w*−1)(*D*_*c*_+*D*_*h*_)−*z*[(α−1)*w*+1]*D*_*c*_ < 0, the stability analysis of the equilibrium points in the replication dynamic system is summarized in Table 4.

**Table 4:** Analysis of the asymptotic stability of the equilibrium point of replication dynamic systems under loose government regulation.

**Equilibrium point**	**Characteristic value λ_1_, λ_2_, λ_3_, λ_4_**	**Positive and negative**	**Stability situation**	**Stability**
(0,0,0,0)	λ_1_ = *D*_*c*_+*D*_*h*_−*C*_*g*_ λ_2_ = −*C*_*h*_−*S*_*h*_ λ_3_ = −*C*_*c*_−*S*_*c*_ λ_4_ = −*C*_*p*_	×−−	*D*_*c*_+*D*_*h*_−*C*_*g*_ < 0	ESS
(0,0,0,1)	λ_1_ = α(*D*_*c*_+*D*_*h*_)−*C*_*g*_+*k*_*g*_ λ_2_ = −*C*_*h*_−*S*_*h*_+2*k*_*h*_ λ_3_ = −*C*_*c*_−*S*_*c*_+2*k*_*c*_ λ_4_ = *C*_*p*_	× × × +	*Have a positive characteristic root*.	*Unstable point*
(0,0,1,0)	λ_1_ = *D*_*h*_−*C*_*g*_ λ_2_ = −*C*_*h*_−*S*_*h*_ λ_3_ = *C*_*c*_+*S*_*c*_ λ_4_ = −*C*_*p*_	×−+−	*Have a positive characteristic root*.	*Unstable point*
(0,0,1,1)	λ_1_ = α*D*_*h*_−*C*_*g*_+*k*_*g*_ λ_2_ = −*C*_*h*_−*S*_*h*_+2*k*_*h*_ λ_3_ = *C*_*c*_+*S*_*c*_−2*k*_*c*_ λ_4_ = *C*_*p*_	× × × +	*Have a positive characteristic root*.	*Unstable point*
(0,1,0,0)	λ_1_ = *D*_*c*_−*C*_*g*_ λ_2_ = *C*_*h*_+*S*_*h*_ λ_3_ = −*C*_*c*_−*S*_*c*_ λ_4_ = −*C*_*p*_	×+−	*Have a positive characteristic root*.	*Unstable point*
(0,1,0,1)	λ_1_ = α*D*_*c*_−*C*_*g*_+*k*_*g*_ λ_2_ = *C*_*h*_+*S*_*h*_−2*k*_*h*_ λ_3_ = −*C*_*c*_−*S*_*c*_+2*k*_*c*_ λ_4_ = *C*_*p*_	× × × +	*Have a positive characteristic root*.	*Unstable point*
(0,1,1,0)	λ_1_ = −*C*_*g*_−*R*_*c*_−*R*_*h*_ λ_2_ = *C*_*h*_+*S*_*h*_ λ_3_ = *C*_*c*_+*S*_*c*_ λ_4_ = −*C*_*p*_	−++−	*Have a positive characteristic root*.	*Unstable point*
(0,1,1,1)	λ_1_ = 2*k*_*g*_−*R*_*c*_−*R*_*h*_−*C*_*g*_ λ_2_ = *C*_*h*_+*S*_*h*_−2*k*_*h*_ λ_3_ = *C*_*c*_+*S*_*c*_−2*k*_*c*_ λ_4_ = *C*_*p*_	× × × +	*Have a positive characteristic root*.	*Unstable point*

As shown in Table 4, in the case of loose regulation by the government, there is no stable strategy that can effectively achieve medical carbon neutrality. This is because the government's loose regulation strategy cannot effectively restrain the improper behavior of public hospitals and pharmaceutical enterprises. To avoid this, the government should increase the punishment for public hospitals that adopt weak enforcement strategies and pharmaceutical enterprises that choose not self-discipline strategies. Simultaneously, the government should also reduce its regulatory costs through relevant measures. Therefore, government must increase penalties for public hospitals adopting weak enforcement strategies and pharmaceutical enterprises employing not self-discipline strategies, while reducing their regulatory costs, to achieve medical carbon neutrality.

### 5.2 Asymptotic stability analysis of the equilibrium points of replication dynamic systems under strict government regulation

During the implementation of medical carbon neutrality strategies, when the government's stable strategy is strict regulation, that is, when the following condition is satisfied: *k*_*g*_−*C*_*g*_−*y*[(α*w*−*w*+1)*D*_*h*_+*z*(*R*_*c*_+*R*_*h*_−*wC*_*g*_)]−(*w*−α*w*−1)(*D*_*c*_+*D*_*h*_)−*z*[(α−1)*w*+1]*D*_*c*_>0, the stability analysis of the equilibrium points in the replication dynamic system is summarized inTable 5.

**Table 5:** Analysis of the asymptotic stability of the equilibrium point of replicating dynamic systems under strict government regulation.

**Equilibrium point**	**Characteristic value λ_1_**, **λ_2_**, **λ_3_**, **λ_4_**	**Positive and negative**	**Stability situation**	**Stability**
(1,0,0,0)	λ_1_ = *C*_*g*_−*D*_*c*_−*D*_*h*_ λ_2_ = *D*_*h*_−*C*_*h*_−*S*_*h*_ λ_3_ = *D*_*c*_−*C*_*c*_−*S*_*c*_ λ_4_ = *D*_*c*_−*C*_*p*_+*D*_*h*_−α(*D*_*c*_+*D*_*h*_)	× × × ×	*C*_*g*_−*D*_*c*_−*D*_*h*_ < 0 *D*_*h*_−*C*_*h*_−*S*_*h*_ < 0 *D*_*c*_−*C*_*c*_−*S*_*c*_ < 0 *D*_*c*_−*C*_*p*_+*D*_*h*_−α(*D*_*c*_+*D*_*h*_) < 0	*ESS*
(1,0,0,1)	λ_1_ = *C*_*g*_−α(*D*_*c*_+*D*_*h*_)−*k*_*g*_ λ_2_ = *D*_*h*_−*C*_*h*_−*S*_*h*_+2*k*_*h*_ λ_3_ = *D*_*c*_−*C*_*c*_−*S*_*c*_+2*k*_*c*_ λ_4_ = −*D*_*c*_+*C*_*p*_−*D*_*h*_+α(*D*_*h*_+*D*_*c*_)	× × × ×	*C*_*g*_−α(*D*_*c*_+*D*_*h*_)+*k*_*g*_ < 0 *D*_*h*_−*C*_*h*_−*S*_*h*_+2*k*_*h*_ < 0 *D*_*c*_−*C*_*c*_−*S*_*c*_+2*k*_*c*_ < 0 −*D*_*c*_+*C*_*p*_−*D*_*h*_+α(*D*_*c*_+*D*_*h*_) < 0	*ESS*
(1,0,1,0)	λ_1_ = *C*_*g*_−*D*_*h*_ λ_2_ = *D*_*h*_−*C*_*h*_+*R*_*h*_−*S*_*h*_ λ_3_ = −*D*_*c*_+*C*_*c*_+*S*_*c*_ λ_4_ = (1−α)*D*_*h*_−*C*_*p*_	× × × ×	*C*_*g*_−*D*_*h*_ < 0 *D*_*h*_−*C*_*h*_−*S*_*h*_+*R*_*h*_ < 0 −*D*_*c*_+*C*_*c*_+*S*_*c*_−2*k*_*c*_ < 0 (α−1)*D*_*h*_+*C*_*p*_ < 0	*ESS*
(1,0,1,1)	λ_1_ = *C*_*g*_−α*D*_*h*_−*k*_*g*_ λ_2_ = *D*_*h*_−*C*_*h*_−*S*_*h*_+*R*_*h*_+2*k*_*h*_ λ_3_ = −*D*_*c*_+*C*_*c*_+*S*_*c*_−2*k*_*c*_ λ_4_ = (1−α)*D*_*h*_+*C*_*p*_	× × × ×	*C*_*g*_−α*D*_*h*_−*k*_*g*_ < 0 *D*_*h*_−*C*_*h*_−*S*_*h*_+*R*_*h*_+2*k*_*h*_ < 0 −*D*_*c*_+*C*_*c*_+*S*_*c*_−2*k*_*c*_ < 0 (α−1)*D*_*h*_+*C*_*p*_ < 0	*ESS*
(1,1,0,0)	λ_1_ = *C*_*g*_−*D*_*c*_ λ_2_ = −*D*_*h*_+*C*_*h*_+*S*_*h*_ λ_3_ = *D*_*c*_−*C*_*c*_−*S*_*c*_+*R*_*c*_ λ_4_ = (1−α)*D*_*h*_−*C*_*p*_	× × × ×	*C*_*g*_−*D*_*c*_ < 0 −*D*_*h*_+*C*_*h*_+*S*_*h*_ < 0 *D*_*c*_−*C*_*c*_−*S*_*c*_+*R*_*c*_ < 0 (1−α)*D*_*h*_−*C*_*p*_ < 0	*ESS*
(1,1,0,1)	λ_1_ = *C*_*g*_−α*D*_*c*_−*k*_*g*_ λ_2_ = −*D*_*h*_+*C*_*h*_+*S*_*h*_−2*k*_*h*_ λ_3_ = *D*_*c*_−*C*_*c*_−*S*_*c*_+*R*_*c*_+2*k*_*c*_ λ_4_ = (α−1)*D*_*h*_+*C*_*p*_	*× × × × *	*C*_*g*_−α*D*_*c*_−*k*_*g*_ < 0 −*D*_*h*_+*C*_*h*_+*S*_*h*_−2*k*_*h*_ < 0 *D*_*c*_−*C*_*c*_−*S*_*c*_+*R*_*c*_+2*k*_*c*_ < 0 (α−1)*D*_*h*_+*C*_*p*_ < 0	*ESS*
(1,1,1,0)	λ_1_ = *C*_*g*_+*R*_*c*_+*R*_*h*_ λ_2_ = −*D*_*h*_+*C*_*h*_+*S*_*h*_−*R*_*h*_ λ_3_ = −*D*_*c*_−*R*_*c*_+*C*_*c*_+*S*_*c*_ λ_4_ = −*C*_*p*_	*+ × × −*	*Have a positive characteristic root*.	*Unstable point*
(1,1,1,1)	λ_1_ = *R*_*c*_+*R*_*h*_+*C*_*g*_−2*k*_*g*_ λ_2_ = −*D*_*h*_+*C*_*h*_+*S*_*h*_−*R*_*h*_−2*k*_*c*_ λ_3_ = *C*_*c*_−*D*_*c*_−*R*_*c*_+*S*_*c*_−2*k*_*c*_ λ_4_ = *C*_*p*_	× × × +	*Have a positive characteristic root*.	*Unstable point*

From Table 5, it can be seen that under the strict regulation of government, there are 6 possible stable strategy combinations, namely (1, 0, 0, 0), (1, 0, 0, 1), (1, 0, 1, 0), (1, 0, 1, 1), (1, 1, 0, 0), (1, 1, 0, 1); There are 3 stable strategy combinations of public participation (1, 0, 0, 1), (1, 0, 1, 1), (1, 1, 0, 1). When the public participates and the government chooses strict regulation, pharmaceutical enterprises will adopt self-discipline strategies to avoid excessive losses; public hospitals will tend to adopt a weak enforcement strategy due to the strategic choice of the other three players in the game. In this scenario, the strategy combination (1, 0, 1, 1) becomes the evolutionarily stable policy combination of the system. However, even when government strictly regulates, the public participates, and public hospitals enforce rigorously, some pharmaceutical enterprises may still choose not self-discipline strategies in pursuit of excessive profits, which is the underlying reason why the strategy combination (1, 1, 0, 1) can become a stable strategy. To prevent (1, 1, 0, 1) from stabilizing, the government must significantly increase penalties for pharmaceutical enterprises adopting not self-discipline strategies. Simultaneously, consumers should also actively participate in public opinion supervision of pharmaceutical enterprises to ensure the smooth realization of carbon neutrality in the medical industry. We can get some enlightenment from the observation of these stable equilibrium points: to safeguard public health rights and interests for a long time and promote the green transformation of the medical industry, government must enforce effective supervision over public hospitals and pharmaceutical enterprises to ensure their proactive engagement in medical carbon neutrality initiatives. The government must invest corresponding regulatory resources to monitor the progress of these key subjects in reducing carbon emissions and improving energy efficiency. Furthermore, the government's strict regulation of public hospitals and pharmaceutical enterprises can effectively curb inefficient or dishonest emission reduction behaviors and prevent them from becoming an asymptotic stability strategy. In the numerical simulations section, further in-depth analysis will be conducted on how government regulation affects the decision-making of public hospitals and pharmaceutical enterprises, and how these behaviors jointly promote the development of the medical industry toward high-quality and low-carbon emissions, to ensure the smooth realization of medical carbon neutrality.

## 6 Numerical simulations

To effectively illustrate the influence of critical parameters in the replicator dynamic system on the evolutionary processes and results of multi-agent games, we use MATLAB 2021b to conduct numerical simulations of each agent's evolutionary paths.

Considering the research hypothesis of this article, based on evolutionary stable strategies (strict government regulation, weak enforcement in public hospitals, self-regulation of pharmaceutical companies, and public participation) and convergence conditions (*C*_*g*_−α*D*_*h*_−*k*_*g*_ < 0,*D*_*h*_−*C*_*h*_−*S*_*h*_+*R*_*h*_+2*k*_*h*_ < 0,−*D*_*c*_+*C*_*c*_+*S*_*c*_−2*k*_*c*_ < 0,(α−1)*D*_*h*_+*C*_*p*_ < 0). And combined with the real situation, we have made initial settings for the model parameters, as follows:

The government regulatory constraint conditions *C*_*g*_−α*D*_*h*_−*k*_*g*_ < 0 indicates that in the context of public participation, the increase in government credibility *k*_*g*_ and the sum of fines α*D*_*h*_ paid by public hospitals under non-self regulatory strategies are greater than the cost *C*_*g*_ paid by the government in strict regulation. In other words, the net benefit of strict regulation is higher than that of loose regulation, so the government is more inclined to adopt strict regulation. Based on this, we set *k*_*g*_ = 5,*C*_*g*_ = 6.

The constraint condition *D*_*h*_−*C*_*h*_−*S*_*h*_+*R*_*h*_+2*k*_*h*_ < 0 for the implementation strategy of public hospitals indicates that under strict government supervision, if public hospitals adopt weak implementation of medical carbon neutrality policies, their short-term benefits *S*_*h*_ minus the fines paid *D*_*h*_ are greater than the benefits obtained when adopting strong implementation strategies *R*_*h*_ plus the credibility gained through public participation 2*k*_*h*_, minus the cost of strong implementation strategies *C*_*g*_. At this point, the net profit from weak execution is higher than that from strong execution, and hospitals tend to lean toward weak execution. According to constraints *C*_*g*_−α*D*_*h*_−*k*_*g*_ < 0 and *D*_*h*_−*C*_*h*_−*S*_*h*_+*R*_*h*_+2*k*_*h*_ < 0, we set *D*_*h*_ = 6,*C*_*h*_ = 4,*S*_*h*_ = 10,*R*_*h*_ = 2,*k*_*h*_ = 2.

The constraint condition −*D*_*c*_+*C*_*c*_+*S*_*c*_−2*k*_*c*_ < 0 for the self-discipline strategy of pharmaceutical enterprises indicates that under strict government regulation, if pharmaceutical enterprises adopt non-self-discipline strategies, their short-term benefits *S*_*c*_ minus fines *D*_*c*_ are less than the credibility obtained under self-discipline strategies 2*k*_*h*_ minus self-discipline costs *C*_*c*_. Therefore, the net benefits of self-discipline are higher, and companies tend to lean toward self-discipline. Based on this, we set *D*_*c*_ = 6, *C*_*c*_ = 3, *S*_*c*_ = 5, *k*_*c*_ = 6.

The constraint condition for public participation (α−1)*D*_*h*_+*C*_*p*_ < 0 indicates that under strict government regulation, the cost of public participation *C*_*g*_ is less than the compensation received, resulting in a higher net benefit of participation than non-participation. Based on this and combining *C*_*g*_−α*D*_*h*_−*k*_*g*_ < 0 with this condition, we set *C*_*p*_ = 1,α = 0.5. In addition, according to reference (54), in the field of environmental protection, the government's benefits *R*_*g*_ under reasonable regulation are greater than its regulatory costs *C*_*g*_, and therefore *R*_*g*_ = 10 is set. The conclusion of reference (55) is that when corporate self-regulation is combined with strict government regulation, the benefits *R*_*c*_ obtained are greater than the self-regulation costs *C*_*c*_. Based on this, *R*_*c*_ = 4 is set. The above settings are shown in Table 6.

**Table 6:** Parameter value.

**Constrained condition**	**Meaning explanation**	**Derive logic**	**Parameter setting**
*C*_*g*_−α*D*_*h*_−*k*_*g*_ < 0	Under public participation, the sum of the government's credibility gain *k*_*g*_ and the fine paid by hospitals adopting a weak enforcement strategy α*D*_*h*_ exceeds the cost of strict regulation *C*_*g*_. Thus, the net benefit of strict regulation is higher than that of loose regulation.	The government tends to adopt strict regulation.	*k*_*g*_ = 5, *C*_*g*_ = 6
*D*_*h*_−*C*_*h*_−*S*_*h*_+*R*_*h*_+2*k*_*h*_ < 0	For public hospitals, the net benefit of adopting a weak enforcement strategy is higher than that of adopting a strong enforcement strategy under strict government regulation.	Public hospitals tend to adopt weak enforcement.	*D*_*h*_ = 6, *C*_*h*_ = 4, *S*_*h*_ = 10,*R*_*h*_ = 2, *k*_*h*_ = 2
−*D*_*c*_+*C*_*c*_+*S*_*c*_−2*k*_*c*_ < 0	For pharmaceutical enterprises, the net benefit of adopting a self-discipline strategy is higher than that of adopting a no self-discipline strategy under strict government regulation.	Pharmaceutical enterprises tend to adopt self-discipline	*D*_*c*_ = 6, *C*_*c*_ = 3, *S*_*c*_ = 5, *k*_*c*_ = 6.
(α−1)*D*_*h*_+*C*_*p*_ < 0	The net benefit of public participation is higher than that of non-participation under strict government regulation.	The public tends to participate.	*C*_*p*_ = 1, α = 0.5
*R*_*g*_>*C*_*g*_ (54)	The government's revenue from reasonable regulation in the environmental protection sector is greater than its regulatory cost.	Regulatory revenue setting.	*R*_*g*_ = 10
*R*_*c*_>*C*_*c*_ (55)	The revenue obtained when corporate self-discipline is combined with strict government regulation exceeds the cost of self-discipline.	Comprehensive enterprise revenue setting.	*R*_*c*_ = 4

Finally, in order to observe how the dynamic process of evolutionary game deviates from equilibrium due to parameter influence, we set the initial strategy probabilities of each player to *x* = 0.5, *y* = 0.5, *z* = 0.5, *w* = 0.5; And numerical simulation was conducted through 100 iterations.

### 6.1 Impact of the cost of strict government supervision

**Figure 6:**
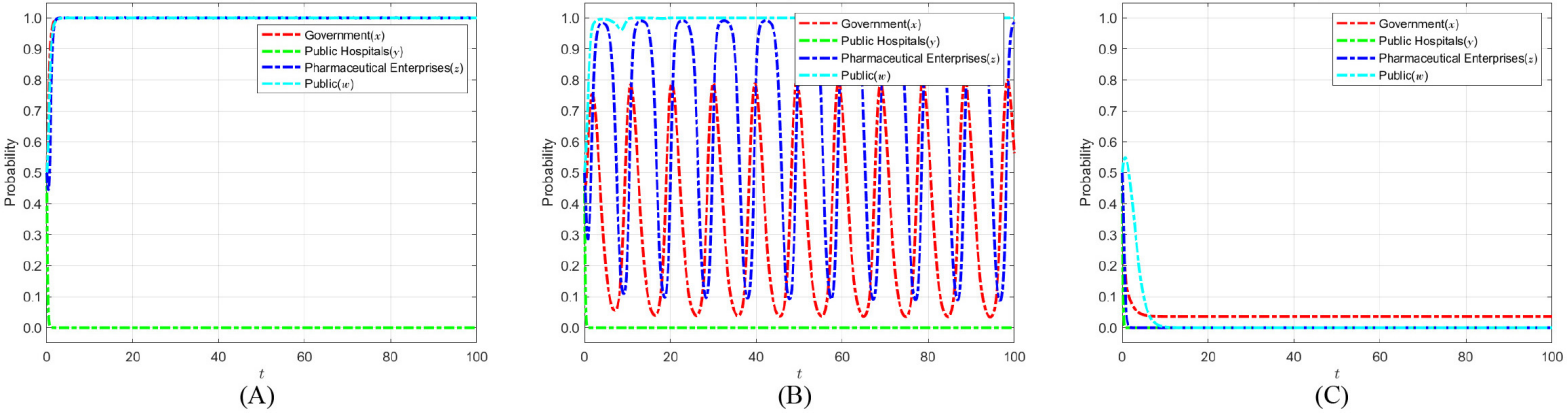
The impact of strict supervisory costs *C*_*g*_ on the evolution of strategies of various parties. **(A)** *C*_*g*_ = 6. **(B)** *C*_*g*_ = 9. **(C)** *C*_*g*_ = 12.

The cost of strict government supervision (*C*_*g*_) was set to 6, 9, and 12 to examine the sensitivity of stakeholders' strategic evolution to changes in regulatory expenditure. These values were chosen to span low, medium, and high-cost scenarios, ensuring comparability with other parameters of similar scale as in the initial settings. Such a range enables the identification of threshold effects predicted by the theoretical analysis, particularly the transition between strict and loose regulatory strategies. The selection also facilitates intuitive visualization of evolutionary trajectories and supports reproducibility of results under varying cost conditions. Figure 6 shows the evolution process and results of the strategies of the main players in the four-party game.

Figure 6 shows that as the cost of strict government regulation increases, the probability of the government adopting a strict regulation approach exhibits a consistently declining trend, ultimately stabilizing at 0. This phenomenon illustrates that, due to severe cost restrictions, the government's capacity for strict regulation diminishes, making it difficult to maintain consistent and successful regulatory initiatives. Simultaneously, as the likelihood of government regulation diminishes, the probability of public engagement in its capacity as a “watchdog” exhibits an increasing tendency, nearing 1 during the stabilization period. This implies that when the public perceives that the government is becoming less inclined toward strict regulation, they will compensate for the regulatory deficit by increasing their participation. Thus, establishing collective supervision of the medical carbon neutrality process. However, when the cost of government regulation rises to the point where the public believes that the government can no longer adopt strict regulatory strategies, the motivation for active public participation will sharply decrease and eventually stabilize at 0. The “synergistic failure” impact indicates that the implementation of carbon neutrality in the healthcare industry will be significantly obstructed if both governmental regulatory willingness and public participation are minimal. Consequently, the government ought to concentrate on refining the design and execution of the regulatory framework, while minimizing regulatory costs through enhanced efficiency, judicious resource allocation, and the incorporation of technological solutions. Simultaneously, it should establish a multi-faceted incentive structure and fortify institutionalized avenues for public engagement and feedback loops, thereby augmenting public motivation for ongoing oversight. Only with the dual drive to reduce regulatory costs and activate public participation can a stable synergistic evolution between the government and the public be formed. This, in turn, enables the successful realization of carbon neutrality in healthcare and provides a replicable empirical model for public health governance.

### 6.2 The impact of government social credibility on the strategic choices of various parties

**Figure 7:**
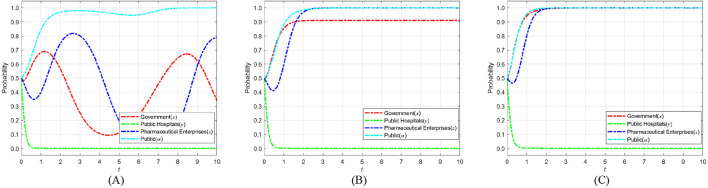
The impact of government social credibility *k*_*g*_ on the strategic choices of various parties. **(A)** *k*_*g*_ = 1. **(B)** *k*_*g*_ = 3. **(C)** *k*_*g*_ = 5.

To investigate how government credibility affects strategic evolution, we set the added value of public credit *k*_*g*_ to 1, 3, and 5 based on initial parameters. When *k*_*g*_ = 1, strict regulation brings only a small credibility gain. This tests the stability of strategies under weak public endorsement. When *k*_*g*_ = 3, the credibility gain is moderate. This reflects common real-world cases and captures the transition from low to high credibility. When *k*_*g*_ = 5, strict regulation greatly boosts public trust. This represents a strong public support scenario. These settings allow an analysis of how different credibility levels shape the evolutionary paths of the government, public hospitals, pharmaceutical enterprises, and the public in achieving medical carbon neutrality. Figure 7 shows the evolution process and results of the strategies of the main players in the four-party game.

Figure 7 illustrates that as public trust in the government's strict regulatory approach increases during the medical carbon neutralization process, the system's evolutionary dynamics exhibit a pronounced “two-way enhancement” effect: firstly, the likelihood of the government adopting a strict regulatory strategy rises consistently with increasing social credibility, ultimately approaching 1; secondly, the probability of pharmaceutical enterprises implementing self-discipline strategies also escalates rapidly in the high-pressure regulatory environment, driven by a substantial rise in not self-discipline costs, eventually stabilizing at 1. This is because as public trust increases, the marginal benefit of the government's choice of strict regulation continuously exceeds that of loose regulation, which not only effectively safeguards the public interest, but also enhances the legitimacy of regulation with the support of public opinion. Conversely, when strict government regulation becomes the dominant strategy, pharmaceutical enterprises that choose the not self-regulation strategy will face higher risks of penalties and reputational losses, and their expected returns are significantly lower than those of the self-discipline strategy, thus tilting toward self-discipline in their strategy evolution. Consequently, improving the public's social credibility in government regulators is a crucial impetus for encouraging the government to proactively implement a strict regulatory strategy. Additionally, it is essential to enhance information transparency and societal regulation of the regulatory process, refine the public participation evaluation system, and utilize public opinion to inform and incentivize mechanisms that promote self-discipline among pharmaceutical enterprises. This ensures that the goal of healthcare carbon neutrality is efficiently achieved through the evolution of the four-party interaction between the government, public hospitals, pharmaceutical companies and the public.

### 6.3 The impact of short-term financial profit on the strategic choices of various parties

**Figure 8:**
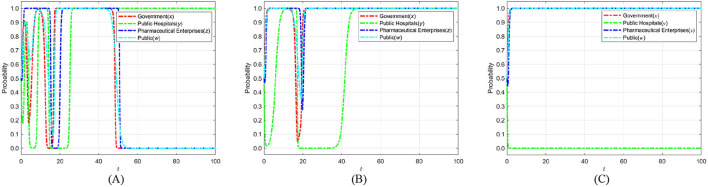
The impact of short-term financial profit *S*_*h*_ on the strategic choices of various parties. **(A)** *S*_*h*_ = 4. **(B)** *S*_*h*_ = 7. **(C)** *S*_*h*_ = 10.

In our model, the tendency of public hospitals to choose weak enforcement is governed by *D*_*h*_−*C*_*h*_−*S*_*h*_+*R*_*h*_+2*k*_*h*_ < 0 and *C*_*g*_−α*D*_*h*_−*k*_*g*_ < 0 The former condition reflects that when the short-term revenue *S*_*h*_ of public hospitals is high, after deducting the fine *D*_*h*_ and the cost *C*_*h*_ under the strong enforcement strategy, their net revenue may still exceed the sum of the direct revenue *R*_*h*_ and reputation gain *k*_*h*_ brought by strong enforcement, thereby prompting hospitals to lean toward weak enforcement; The latter condition ensures that the government still chooses strict regulation when the regulatory benefits outweigh the costs. Based on this, we set *S*_*h*_ to 4, 7, and 10, representing low, medium, and high short-term return scenarios, respectively, covering three typical states of not meeting, approaching, and significantly meeting weak enforcement conditions. This facilitates sensitivity analysis and critical effect identification, to better analyze the evolutionary path of the four party game subjects in the process of implementing medical carbon neutrality. Figure 8 shows the evolution process and results of the strategies of the main players in the four-party game.

The evolutionary trajectory depicted in Figure 8 illustrates that as the short-term benefits of public hospitals adopting weak enforcement strategies for medical carbon neutrality policies rise the probability of their inclination toward strong enforcement strategies diminishes. This ultimately results in evolutionary stability with weak enforcement strategies. During this process, a dual compensation mechanism is established within the system: “weak enforcement by public hospitals necessitates strict government regulation, and strict government regulation fosters heightened public participation.” On one hand, the rising likelihood of weak enforcement by public hospitals jeopardizes the public interest in healthcare carbon emission management, prompting the government to adopt a strict regulatory strategy, ultimately stabilizing the probability of its regulation at 1. On the other hand, the public, after observing the increased willingness of the government to regulate, correspondingly increased their motivation to participate in social supervision, and their probability of participation eventually stabilized at 1 as well. At the same time, under the dual pressure of government regulation and public opinion, pharmaceutical enterprises are experiencing a reversal in the cost-benefit structure of self-discipline enforcement, and the probability of adopting self-discipline strategies is gradually approaching 1. The outcomes of the aforementioned evolutionary game suggest that the government should implement precise incentive and constraint policies. These policies aim to tighten the short-term revenue space of public hospitals' weak performance. This also can be achieved through financial incentives, performance evaluations, and other measures. Simultaneously, the government and the public should strengthen their joint oversight of pharmaceutical enterprises downstream in the healthcare supply chain. This can be achieved through information disclosure, third-party evaluation, and other means. Only under the triple driving force of weakening the short-term interests of hospitals, strengthening the linkage between government and the public, and guiding the self-discipline of enterprises can the synergistic evolution system of carbon neutrality in the healthcare industry be firmly built. This, in turn, ensures that the goal of carbon neutrality is achieved on schedule.

### 6.4 The impact of penalty fines on the strategic choices of various parties when the pharmaceutical enterprise selects no self-discipline

**Figure 9:**
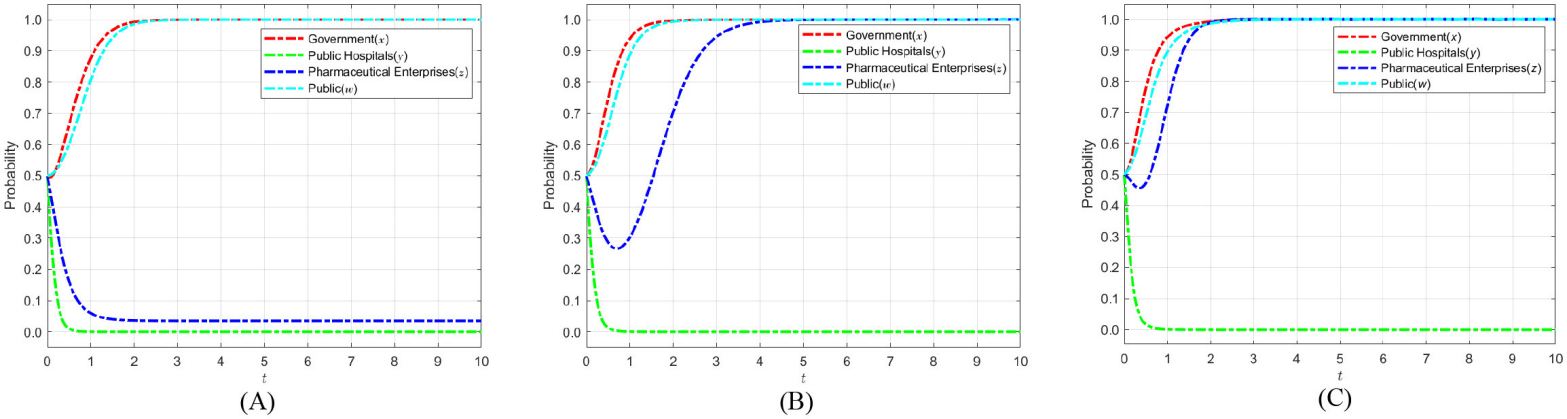
The impact of penalty fine *D*_*c*_ on the strategic choices of various parties when the pharmaceutical enterprise selects no self-discipline. **(A)** *D*_*c*_ = 2. **(B)** *D*_*c*_ = 4. **(C)** *D*_*c*_ = 6.

Based on the constraint −*D*_*c*_+*C*_*c*_+*S*_*c*_−2*k*_*c*_ < 0, the fines *D*_*c*_ imposed on pharmaceutical enterprises for failing to self-discipline are set at 2, 4, and 6. Different fine levels allow analysis of how penalty intensity influences the evolution of pharmaceutical strategies. Low fines may fail to incentivize self-discipline, while moderate adjustments help identify an effective range that promotes self-regulation, ensuring reasonable and meaningful parameter settings. This approach enables the model to better reflect real regulatory environments and enhances the practical relevance of the results. The evolution process and results of the strategies of the main players in the four-party game are shown in Figure 9.

Figure 9 illustrates that following the government's augmentation of fines for pharmaceutical enterprises employing not self-discipline strategies, the probability of self-discipline among these enterprises will markedly rise, ultimately nearing 1. Consequently, when pharmaceutical enterprises implement not self-discipline tactics, the government can significantly diminish the likelihood of such behavior by increasing fines against pharmaceutical enterprises. This initiative can facilitate the attainment of medical carbon neutrality.

### 6.5 The impact of pharmaceutical enterprises' short-term profit on the strategic choices of various parties

**Figure 10:**
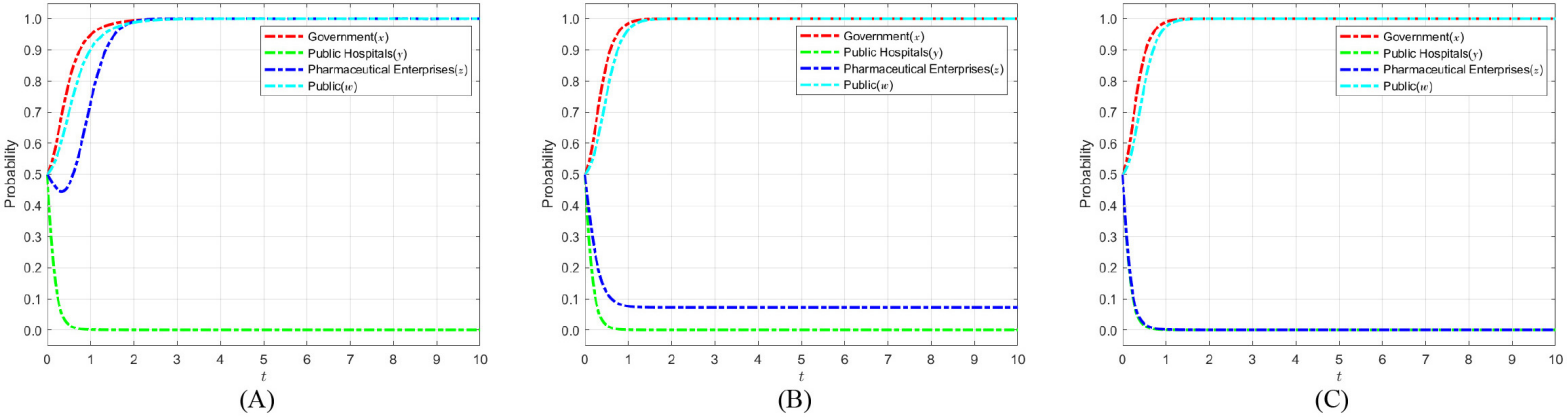
The impact of pharmaceutical enterprises' short-term profit *S*_*c*_ on the strategic choices of various parties. **(A)** *S*_*c*_ = 5. **(B)** *S*_*c*_ = 9. **(C)** *S*_*c*_ = 13.

Based on the constraint −*D*_*c*_+*C*_*c*_+*S*_*c*_−2*k*_*c*_ < 0, the short-term benefit *S*_*c*_ together with fines *D*_*c*_, costs *C*_*c*_, and other regulatory parameters determine the net payoff for pharmaceutical enterprises when they fail to self-discipline. To simulate firms' behavioral responses under different economic incentives, the short-term benefits *S*_*c*_ for non-self-discipline are set at 5, 9, and 13. These values cover a range from low to high economic gains, reflecting possible benefit levels under varying policy environments. A lower *S*_*c*_ indicates limited gains from non-compliance, which may not outweigh corresponding penalties and costs, whereas a higher *S*_*c*_ represents greater short-term incentives for firms to choose non-self-regulation. Setting these three levels allows systematic analysis of pharmaceutical enterprises' strategic evolution under different benefit scenarios, revealing the dynamic interplay between policy pressures and firm behavior, and providing theoretical support and strategic guidance for the implementation of carbon neutrality policies. Figure 10 shows the evolution process and results of the strategies of the main players in the four-party game.

As shown in Figure 10, with the increase of short-term benefits of not self-discipline in pharmaceutical enterprises, the benefits of not self-discipline gradually exceed those of self-discipline. This makes pharmaceutical enterprises more interested in pursuing short-term profits, resulting in the probability of pharmaceutical enterprises taking self-discipline strategies gradually decreasing and eventually stabilizing at 0. However, the government maintained strict regulation during this process, and the public participated. Yet this still leads pharmaceutical enterprises to tend toward not self-discipline strategies. The reason is that the government and the public impose insufficient punishment and fail to cause a significant enough loss of reputation when pharmaceutical enterprises adopt no self-discipline. Therefore, the government should take stronger measures to weaken the short-term benefits obtained by pharmaceutical enterprises when they lack self-discipline. Similarly, the public should participate in the supervision process through more channels, strengthening the public opinion pressure on not self-disciplined pharmaceutical enterprises. This will impel pharmaceutical enterprises to adopt self-discipline strategies and participate in implementing medical carbon neutrality strategies.

### 6.6 The impact of the reputation of pharmaceutical enterprises on the strategic choices of various parties

**Figure 11:**
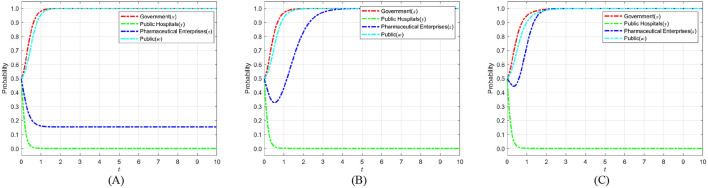
The impact of the reputation of pharmaceutical enterprises *k*_*c*_ on the strategic choices of various parties. **(A)** *k*_*c*_ = 2. **(B)** *k*_*c*_ = 4. **(C)** *k*_*c*_ = 6.

Based on the constraint−*D*_*c*_+*C*_*c*_+*S*_*c*_−2*k*_*c*_ < 0, the credibility enhancement factor *k*_*c*_ plays a critical role in balancing the net payoff for pharmaceutical enterprises. Setting *k*_*c*_ at 2, 4, and 6 allows the model to capture varying levels of credibility incentives that influence firms' strategic behavior. These values represent a gradually increasing scale of credibility reinforcement, which is essential to analyze how changes in the perceived reputation benefits affect the decision-making process of pharmaceutical enterprises. A lower *k*_*c*_ implies weaker credibility incentives, making self-regulation less attractive, while higher values reflect stronger incentives that encourage compliance. By selecting these three representative values, the model can systematically explore the threshold effects of credibility incentives on evolutionary dynamics, providing theoretical support and decision-making references for the implementation of medical carbon neutrality policies. Figure 11 shows the evolution process and results of the strategies of the main players in the four-party game.

As shown in Figure 11, the probability of pharmaceutical enterprises choosing self-discipline increases significantly when public participation confers higher social credibility on these enterprises. This higher credibility encourages them to adopt a self-discipline strategy. Eventually, the probability stabilizes toward 1. Specifically, within the evolutionary game framework, social credibility functions as an external incentive for pharmaceutical enterprises. It drives an increase in the marginal gains they achieve by adopting a self-discipline strategy beyond what they would earn not self-discipline. As an increasing number of pharmaceutical enterprises implement self-discipline tactics, public acknowledgment and social reputation concurrently enhance. This growth in reputation has had a positive feedback effect, further reinforcing the superiority and attractiveness of self-discipline strategies adopted by pharmaceutical enterprises. Based on this, it is advised that the government and industry associations enhance public knowledge regarding the sustainable development accomplishments of pharmaceutical enterprises. And through scientific assessment, self-regulatory commitment disclosure and third-party certification, the public's attention and trust in the green governance practices of pharmaceutical enterprises will be enhanced. On this basis, pharmaceutical enterprises can be guided to transform their accumulated social reputation capital into long-term competitive advantages. This, in turn, enables them to more actively adopt self-discipline strategies in the healthcare carbon-neutralization process and achieve a win-win outcome for both environmental and economic benefits.

### 6.7 The impact of compensation proportion on the strategic choices of various parties

**Figure 12:**
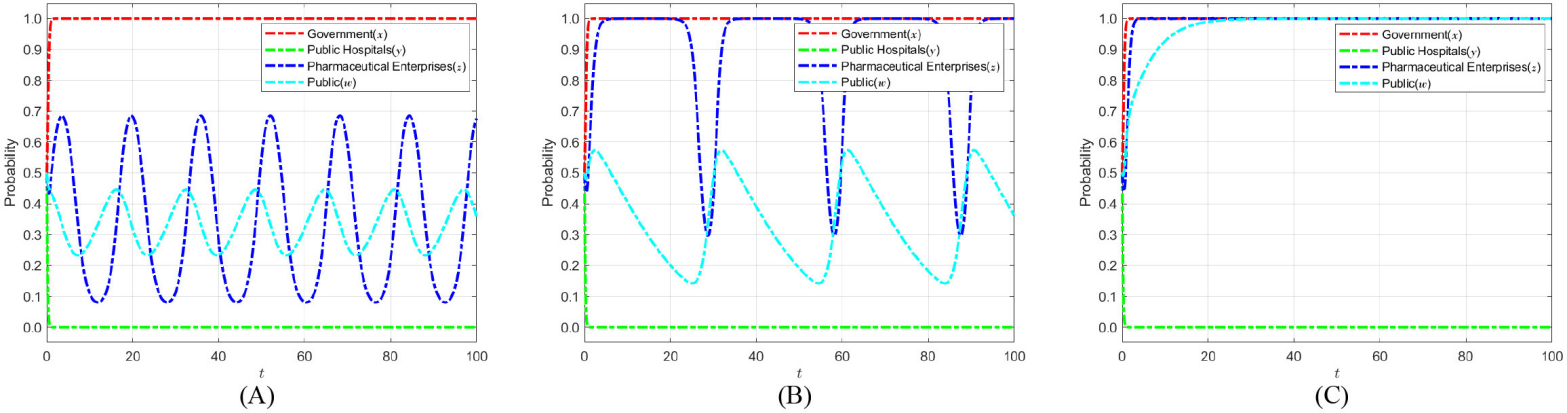
The impact of compensation ratios on the strategic choices of various parties. **(A)** 1 − α= 0.1. **(B)** 1 − α= 0.15. **(C)** 1 − α= 0.2.

The proportion of public compensation 1−α reflects the government's support for public participation in healthcare carbon neutrality and the role of compensation mechanisms in motivating engagement. Based on the constraint (α−1)*D*_*h*_+*C*_*p*_ < 0, the compensation ratio must ensure that the public receives sufficient compensation to offset participation costs *C*_*p*_ and maintain motivation, while avoiding excessive compensation that could lead to resource inefficiency. Therefore, the public compensation ratio is set at 0.1, 0.15, and 0.2, covering a range from low to moderate compensation levels. This range guarantees reasonable returns for the public to encourage active participation in carbon neutrality efforts while maintaining economic sustainability. By establishing these three representative values, the model can effectively reveal how varying compensation levels influence the evolutionary strategies of the public, thereby offering theoretical foundations and policy recommendations for designing compensation mechanisms in healthcare carbon neutrality policies. Figure 12 shows the evolution process and results of the strategies of the main players in the four-party game.

Figure 12 shows that as the compensation proportion for public participation increases, people obtain higher marginal benefits from participating in medical carbon neutrality. Consequently, their participation probability rises significantly and eventually stabilizes at 1. Under the combined effects of increased public participation and strong government regulation, the net benefits pharmaceutical enterprises obtain from a self-discipline strategy begin to exceed those from not self-discipline. This change in benefits compels pharmaceutical enterprises to progressively implement self-discipline measures, ultimately stability at 1. Based on the above analyses, it is suggested that the government should take multi-dimensional measures: on the one hand, it should guide the public to participate in monitoring in a continuous and rational manner by dynamically adjusting the compensation ratio and setting an incentive cap; on the other hand, a third-party assessment organization can be introduced. This organization would publicize the performance of pharmaceutical enterprises in carbon emission reduction and green governance. It could also certify their efforts, thereby strengthening the social credibility effect. In addition, policy tool combinations should be optimized by integrating financial subsidies, tax incentives, and credit endorsements to form a robust benefit–linkage mechanism. This approach will maximize the benefits of public participation and the self-discipline motivation of pharmaceutical enterprises throughout the healthcare carbon-neutralization process, thereby facilitating the achievement of healthcare carbon neutrality.

### 6.8 The impact of public participation costs on the strategic choices of various parties

**Figure 13:**
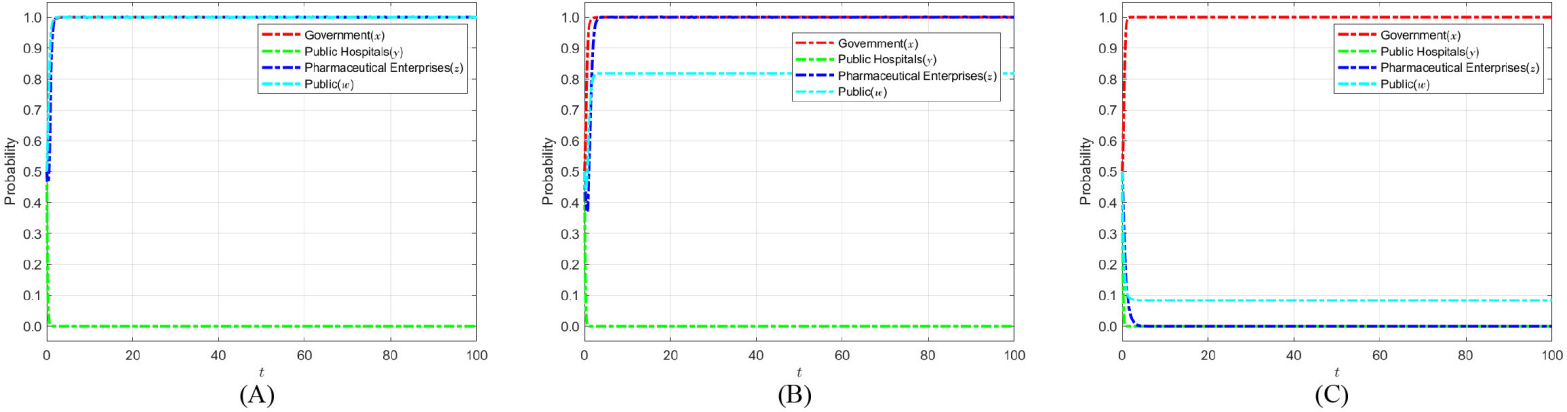
The impact of public participation costs *C*_*p*_ on the strategic choices of various parties. **(A)** *C*_*p*_ = 0. **(B)** *C*_*p*_ = 3. **(C)** *C*_*p*_ = 6.

Based on the constraint (α−1)*D*_*h*_+*C*_*p*_ < 0, there is a trade-off relationship between public participation costs *C*_*p*_ and the public compensation ratio 1−α. To ensure that the public receives adequate compensation to maintain their motivation despite bearing participation costs, these costs need to be set within a reasonable range. Therefore, public participation costs are set at 0, 3, and 6, representing scenarios of no cost, moderate burden, and higher burden, respectively. These values cover various participation barriers that the public may realistically face, facilitating analysis of how changes in costs affect public willingness to participate and their strategic evolution. This layered cost setup, consistent with the constraint, enables the model to scientifically reveal the balance mechanism between compensation and cost, thereby providing theoretical support for designing public participation incentives in healthcare carbon neutrality policies. Figure 13 shows the evolution process and results of the strategies of the main players in the quadripartite game.

Figure 13 illustrates that the probability of public participation in healthcare carbon neutrality markedly decreases as costs associated with participation increase. In this context, due to the lack of public participation, the regulatory responsibility is left only to the government, and the regulatory pressure is insufficient, resulting in a gradual decrease in the probability of pharmaceutical enterprises adopting self-discipline strategies, ultimately stabilizing at adopting not self-discipline strategies. To reverse this trend, the government should enhance public participation by increasing participation compensation, establishing dynamic incentive mechanisms, optimizing participation processes, and reducing time and economic costs. At the same time, third-party review and public opinion monitoring are being introduced to boost social exposure and public pressure on pharmaceutical enterprises that do not self-regulate. The aforementioned actions can improve public oversight and pharmaceutical enterprises' self-discipline, create a multi-party cooperative regulatory force, and contribute in the achievement of medical carbon neutrality.

## 7 Model extension

### 7.1 Analysis of time-delay effect in public hospitals

In this section, we explored the effect of time delays in public hospitals on their evolutionary strategies under evolutionary game theory. We analyzed the influence of time delays on the stability speed of the optimal strategy. To better illustrate the conclusions of this subsection, we introduce parameter τ_*h*_ into the differential equation to represent the time delay effect in the game of public hospitals. The specific model is as follows:


(34) $$\begin{array}{l} \frac{dx\left(t\right)}{dt}=x(t)(1-x(t))\{w(t)k_{g}-C_{g}-y(t-\tau_{h})\\ \;[(\alpha w(t)-w(t)+1)D_{h}+z(t)(R_{c}+R_{h}-w(t)C_{g})]\\ \;-(w(t)-\alpha w(t)-1)(D_{c}+D_{h})-z(t)[(\alpha -1)w(t)+1]D_{c}\} \end{array}$$

(35) $$\begin{array}{l} \frac{dy\left(t\right)}{dt}=y\left(t-\tau_{h}\right)\left[1-y\left(t-\tau_{h}\right)\right]\\ \;\left[x\left(t\right)\left(D_{h}+z\left(t\right)R_{h}\right)+2w\left(t\right)k_{h}-S_{h}-C_{h}\right]\; \end{array}$$

(36) $$\begin{array}{l} \frac{dz\left(t\right)}{dt}=z\left(t\right)\left[1-z\left(t\right)\right]\\ \;\left[x\left(t\right)\left(D_{c}+y\left(t-\tau_{h}\right)R_{c}\right)+2w\left(t\right)k_{c}-S_{c}-C_{c}\right]\; \end{array}$$

(37) $$\begin{array}{l} \frac{dw\left(t\right)}{dt}=w\left(t\right)[1-w(t)]\\ \;\{x(t)(1-\alpha )(D_{c}+D_{h})-x(t)[(1-\alpha )y(t-\tau_{h})D_{h}\\ \;+(1-\alpha )z(t)D_{c}]-C_{p}\} \end{array}$$

**Figure 14:**
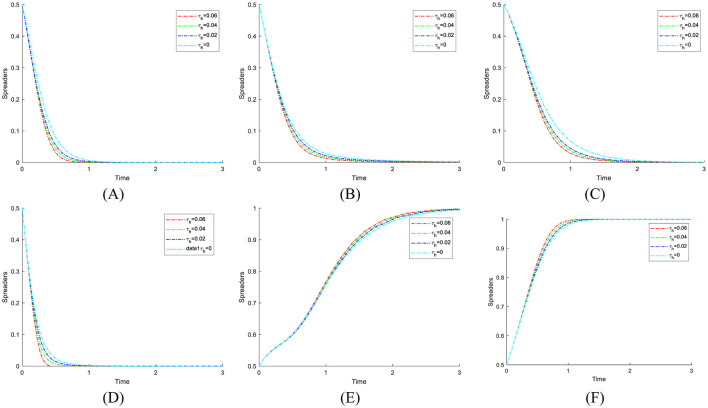
The impact of time delays on public hospitals. **(A)** (1,0,0,0) Stable point. **(B)** (1,0,0,1) Stable point. **(C)** (1,0,1,0) Stable point. **(D)** (1,0,1,1) Stable point. **(E)** (1,1,0,0) Stable point. **(F)** (1,1,0,1) Stable point.

As illustrated in the system of equations, the probability *y* of public hospitals adopting a strong enforcement strategy becomes a function of *t*−τ_*h*_, where τ_*h*_ represents the time delays parameter. To validate this model, six comparative experiments were conducted, with results summarized in Figures 14A–F.

As seen from the above figures, the time delays do not change the strategic evolution direction of public hospitals at different equilibrium points. This indicates that, in the evolutionary game process, time delays do not affect the final strategy selection of public hospitals. Interestingly, as the delay time increases, the speed at which public hospitals converge to the optimal strategy selection becomes faster. This is because, under time delays, the game process among players transforms from a simultaneous static game into a dynamic game with temporal lags. In this context, public hospitals under different equilibrium conditions can observe the strategic selections and stabilization trends of the other three game entities, thereby gaining an informational advantage to adopt strategies that maximize their payoffs. Compared with delay-free scenarios, public hospitals thus stabilize their strategies more rapidly. Notably, with longer delay times, the evolutionary trends of other players become more pronounced and predictable, enabling public hospitals to accelerate their convergence toward the optimal strategy.

Following the time-delay model results, it is notable that such delays—whether arising from policy implementation lags or behavioral inertia—do not destabilize the system. A plausible explanation is that the inherent strategic incentives embedded in the payoff structure are sufficiently robust to preserve equilibrium stability, even when decision-making is temporally misaligned. However, the influence of delays is not necessarily uniform across all stakeholders. For example, government regulators, pharmaceutical enterprises, and the public may respond differently to administrative delays, cognitive delays, or resource allocation delays, leading to varied adjustment speeds and convergence paths. Future research could systematically classify these delay types and investigate their heterogeneous impacts on decision-making dynamics.

From a policy and management perspective, mitigating the adverse effects of delays and enhancing system performance requires a multi-level, cross-sectoral, and integrated coordination framework. First, an adaptive incentive adjustment mechanism based on real-time behavioral feedback should be established. By regularly collecting and analyzing behavioral data from all stakeholders in strategic choices, policy makers can dynamically modify reward and punishment levels to correct deviations in policy implementation. This approach minimizes the risk of incentive obsolescence caused by information lags and ensures that the policy continues to exert a sustained influence on stakeholder behavior. Second, administrative procedures should be streamlined to reduce institutional bottlenecks. This includes clarifying roles and responsibilities at the policy formulation stage, simplifying approval processes, and accelerating cross-departmental information sharing to lower the time cost of policy implementation. Finally, a multi-stakeholder engagement platform should be established, involving government bodies, public hospitals, pharmaceutical enterprises, public representatives, and independent professional institutions. Through regular consultations, transparent information disclosure, and joint decision-making, such a platform can foster early consensus on policy goals and action pathways, thereby reducing behavioral inertia and resistance. These integrated measures can ensure that not only public hospitals but all stakeholders converge toward socially optimal strategies within a reasonable time frame, ultimately enhancing the timeliness, coordination, and sustainability of low-carbon public health policy formulation and implementation.

### 7.2 Analysis of the time-delay effect in pharmaceutical enterprises

In this section, we discuss the impact of time delays on pharmaceutical enterprises' evolutionary strategies in evolutionary games and analyze the influence of time delays on the stability and speed of the optimal strategy. To better illustrate the conclusions of this subsection, we introduce parameter τ_*c*_ into the differential equation to represent the effect of time delays in the game of pharmaceutical enterprises. The specific model is as follows:


(38) $$\begin{array}{l} \frac{dx\left(t\right)}{dt}=x\left(t\right)[1-x\left(t\right)]\{w(t)k_{g}-C_{g}-y(t)[(\alpha w(t)-w(t)+1)\\ \;D_{h}+z(t-\tau_{c})(R_{c}+R_{h}-w(t)C_{g})]-(w(t)-\alpha w(t)-1)\\ \;(D_{c}+D_{h})-z(t-\tau_{c})[(\alpha -1)w(t)+1]D_{c}\}\; \end{array}$$

(39) $$\begin{array}{l} \frac{dy\left(t\right)}{dt}=y\left(t\right)[1-y(t)][x(t)\\ \;\left(D_{h}+z\left(t-\tau_{c}\right)R_{h}\right)+2w(t)k_{h}-S_{h}-C_{h}]\; \end{array}$$

(40) $$\begin{array}{l} \frac{dz\left(t\right)}{dt}=z\left(t-\tau_{c}\right)[1-z(t-\tau_{c})][x(t)\left(D_{c}+y\left(t\right)R_{c}\right)\\ \;+2w(t)k_{c}-S_{c}-C_{c}]\; \end{array}$$

(41) $$\begin{array}{l} \frac{dw\left(t\right)}{dt}=\left(t\right)[1-w(t)]\{x(t)(1-\alpha )(D_{c}+D_{h})-x(t)\\ \;[(1-\alpha )y(t)D_{h}+(1-\alpha )z(t-\tau_{c})D_{c}]-C_{p}\}\; \end{array}$$

**Figure 15:**
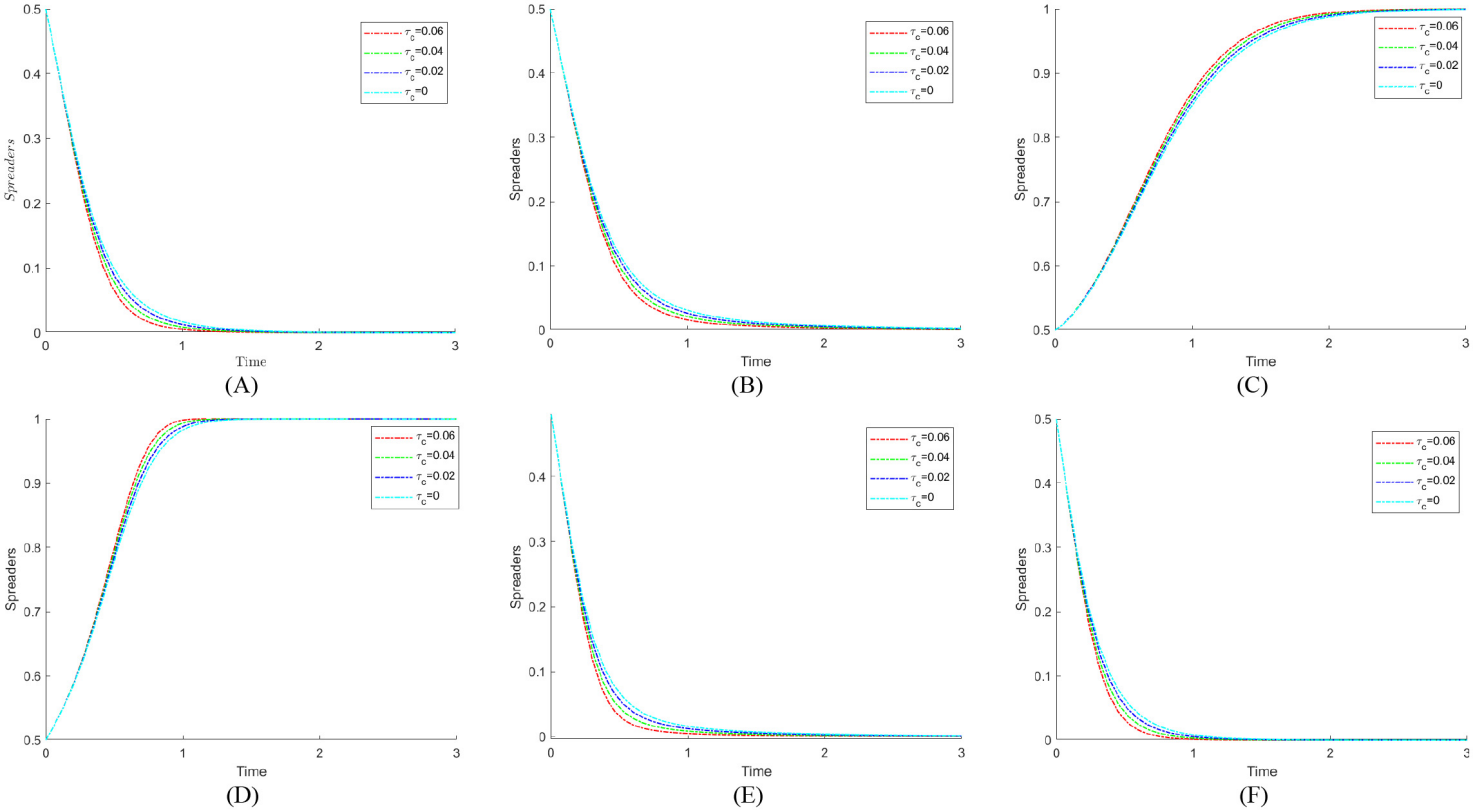
The impact of time delays on pharmaceutical enterprises. **(A)** (1,0,0,0) Stable point. **(B)** (1,0,0,1) Stable point. **(C)** (1,0,1,0) Stable point. **(D)** (1,0,1,1) Stable point. **(E)** (1,1,0,0) Stable point. **(F)** (1,1,0,1) Stable point.

From the above equations, it can be seen that at the not self-discipline probability *z* of pharmaceutical enterprises becomes a function of *t*−τ_*c*_. In order to better illustrate the above model conclusions, six groups of comparative experiments were carried out, as shown in Figures 15A–F.

Figure 15 analysis reveals that time delays do not alter the strategic evolution direction of pharmaceutical enterprises across equilibrium points, indicating that such delays do not affect their final strategy selection in the evolutionary game. Compared to delay-free scenarios, the speed at which pharmaceutical enterprises converge to the optimal strategy increases as delay time lengthens. This acceleration, consistent with the mechanism explained in Section 7.1 for public hospitals, arises from the ability of enterprises to observe and anticipate the stabilization trends of other players before finalizing their own strategy choices.

Notably, although the delay effect does not destabilize the equilibrium, its impact on pharmaceutical enterprises is shaped by industry-specific factors such as R&D investment cycles, regulatory approval timelines, and production capacity adjustments. Different types of delays—such as technology development lags, supply chain disruptions, or market demand recognition delays—may alter the responsiveness of enterprises and, consequently, the system's adaptation speed.

To enhance strategic responsiveness under such delays, pharmaceutical enterprises could focus on strengthening internal early-warning systems to detect market and policy signals in advance, diversifying supply chain structures to reduce vulnerability to disruptions, and employing flexible production planning to shorten adjustment lead times. These enterprise-level strategies complement but differ from system-wide policy measures, targeting the unique operational rhythms and risk structures of the pharmaceutical industry.

### 7.3 Analysis of the time-delay effect in public

In this section, we discuss the impact of time delays on the public's evolutionary strategies in evolutionary games and analyze the influence of time delays on the stability and speed of the optimal strategy. To better illustrate the conclusions of this subsection, we introduce the parameter τ_*p*_ into the differential equation to represent the effect of time delays in the game of the public. The specific model is as follows:


(42) $$\begin{array}{l} \frac{dx\left(t\right)}{dt}=x(t)(1-x(t))\{w(t-\tau_{p})k_{g}-C_{g}-y(t)[(\alpha w(t-\tau_{p})\\ \;-w(t-\tau_{p})+1)D_{h}z(t)(R_{c}+R_{h}-w(t-\tau_{p})C_{g})]\\ \;-[w(t-\tau_{p})-\alpha w(t-\tau_{p})-1)(D_{c}+D_{h})-z(t)[(\alpha 1)\\ \;w(t-\tau_{p})+1]D_{c}\}\; \end{array}$$

(43) $$\begin{array}{l} \frac{dy\left(t\right)}{dt}=y\left(t\right)[1-y(t)][x(t)\left(D_{h}+z\left(t\right)R_{h}\right)\\ \;+2w(t-\tau_{p})k_{h}-S_{h}-C_{h}]\; \end{array}$$

(44) $$\begin{array}{l} \frac{dz\left(t\right)}{dt}=z\left(t\right)[1-z(t)][x(t)\left(D_{c}+y\left(t\right)R_{c}\right)\\ \;+2w(t-\tau_{p})k_{c}-S_{c}-C_{c}]\; \end{array}$$

(45) $$\begin{array}{l} \frac{dw\left(t\right)}{dt}=w\left(t-\tau \right)[1-w(t-\tau_{p})]\{x(t)(1-\alpha )(D_{c}+D_{h})\\ \;-x(t)[\left(1-\alpha \right)y\left(t\right)D_{h}+(1-\alpha )z(t)D_{c}]-C_{p}\}\; \end{array}$$

**Figure 16:**
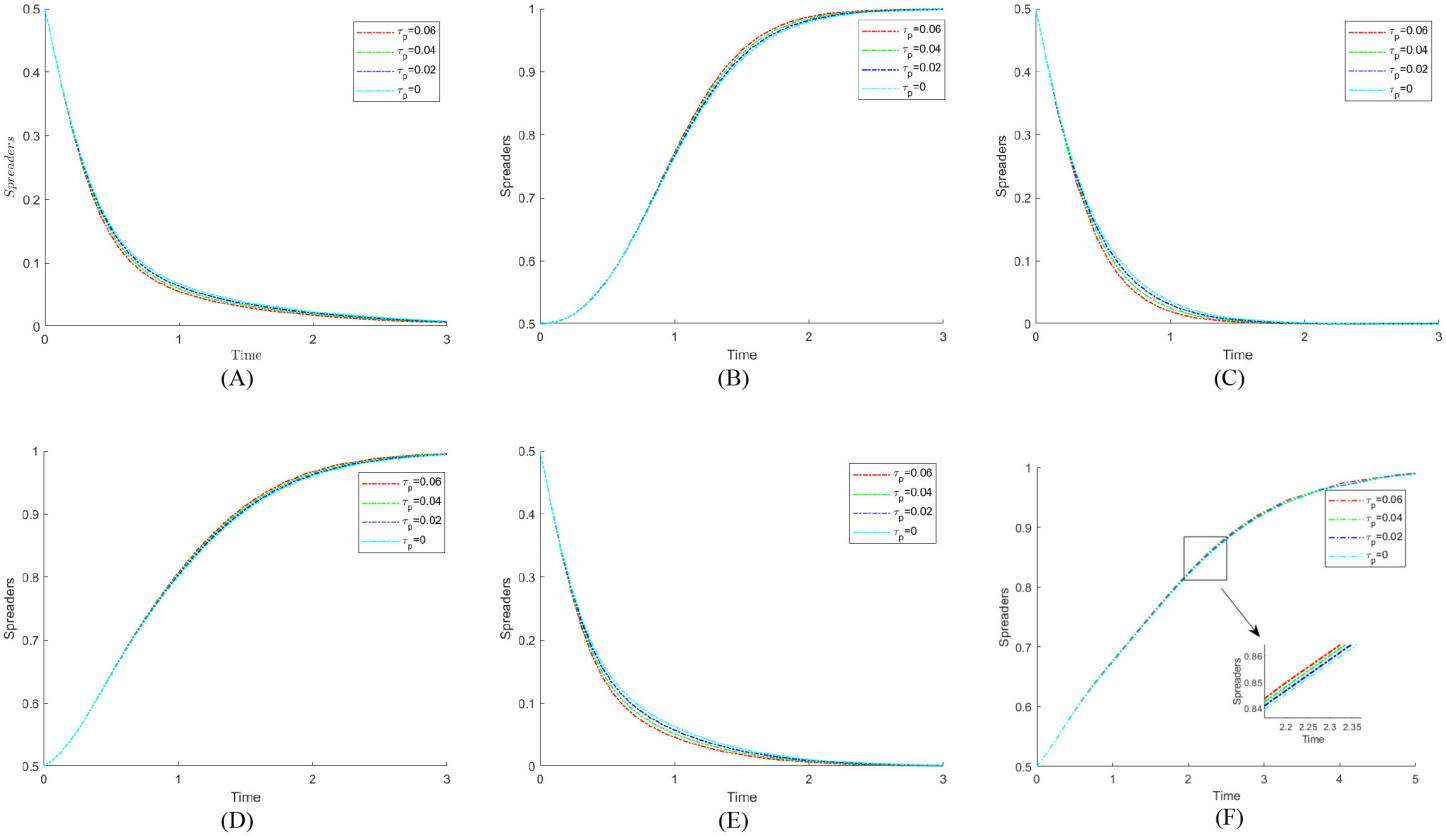
The impact of time delay on the public. **(A)** (1,0,0,0) Stable point. **(B)** (1,0,0,1) Stable point. **(C)** (1,0,1,0) Stable point. **(D)** (1,0,1,1) Stable point. **(E)** (1,1,0,0) Stable point. **(F)** (1,1,0,1) Stable point.

From the above system of equations, it can be observed that the participation probability *w* chosen by public now becomes a function of *t*−τ_*p*_. To better illustrate the above model conclusions, six groups of comparative experiments were carried out, as shown in Figures 16A–F.

The phase diagram analysis indicates that time delays do not alter the evolutionary direction of public strategies across equilibrium points, suggesting that such delays do not influence the ultimate strategic choice of the public in the evolutionary game. However, compared with the delay-free scenario, the public exhibits a faster convergence to the optimal strategy when time delays are present, and this acceleration becomes more pronounced as the delay period increases. This effect may be attributed to the public's enhanced ability to interpret and internalize information signals from other stakeholders during the delayed decision process, thereby reducing hesitation in adopting beneficial strategies. Different forms of delay—such as the dissemination lag of policy information and the adaptation lag in behavioral patterns—may have varying effects on decision-making speed and stability. Addressing these challenges could involve strengthening transparent information channels, improving the timeliness of public engagement campaigns, and facilitating gradual behavioral transitions through staged incentives, so that the public's strategic alignment remains both rapid and robust without destabilizing the system.

## 8 Conclusions

This study systematically analyzed the strategic evolution behavior of the government, public hospitals, pharmaceutical enterprises, and the public in the process of medical carbon neutrality by constructing a four-party evolutionary game model. Numerical simulations were performed using MATLAB 2021b to analyze critical parameters such regulatory costs and credibility, incentives and penalties, public compensation ratios, and participation costs. Time delay effects were incorporated into the model to elucidate the dynamic influence of these factors on the gains and losses of all stakeholders concerned. Considering the aforementioned analysis, we have summarized four critical conclusions that serve as significant references for optimizing carbon neutrality policies within the healthcare sector and improving the efficacy of governmental regulation and public engagement.

(1) The probability of strict government regulation is both constrained by regulatory costs and closely related to government credibility. To promote the effective implementation of carbon neutrality in the healthcare industry, the government should establish and strengthen the regulatory mechanism for energy conservation and emission reduction, promote the adoption of emission reduction strategies in public hospitals, and regulate the behavior of pharmaceutical enterprises. On the one hand, market-oriented means such as carbon trading can be used to reduce the cost of direct government regulation, allowing public hospitals and pharmaceutical enterprises to participate in carbon emission management independently through market trading. On the other hand, the disclosure process of medical carbon neutrality information should be simplified and optimized to improve process efficiency, ensure timely and accurate transmission of supervisory information to the public, and thereby enhance the government's image and credibility. Through the dual drive of marketization and informatization, continuously improving the regulatory system will help promote the smooth implementation of medical carbon neutrality.
(2) The decision-making motives of public hospitals in adopting a weak enforcement strategy are mainly rooted in short-term benefits maximization. Similarly, when choosing a strategy of self-regulation or not self-regulation, pharmaceutical enterprises will weigh not only their short-term gains from not self-regulation, but also the cost of fines that they may face under strict government regulation, as well as the reputational risks associated with public engagement. To this end, the government needs to improve the disciplinary mechanism while building a regulatory system for energy conservation and emission reduction in the healthcare industry. Fines and notification of criticism should be applied to public hospitals that do not execute the healthcare carbon neutralization policy with vigor. For pharmaceutical enterprises that adopt not self-discipline strategies, their institutional costs should be increased by means of revoking their business licenses and incorporating their bad behavior into public credit information platforms. Through a multi-dimensional incentive-constraint mechanism, public hospitals and pharmaceutical enterprises will be promoted to actively fulfill their energy-saving and emission reduction responsibilities. This will ensure that the healthcare carbon-neutrality target is achieved.
(3) Public participation is of great significance in the carbon neutrality process of the healthcare industry and is influenced by both compensation ratios and participation costs. On the one hand, active public participation enhances the green growth of the healthcare industry and increases the relevance and efficacy of government energy conservation and emission reduction programs. On the other hand, the public plays a key role in supervising the behavior of public hospitals and pharmaceutical companies, and their public opinion and reputation leverage can motivate industry entities to continue implementing energy-saving and emission reduction measures. In order to facilitate the green transformation of the medical and health sector, the government should moderately raise the compensation ratio and make full use of digital tools to expand low-cost participation channels like online consultation, electronic questionnaires, and online forums.
(4) Even though public hospitals, pharmaceutical enterprises, and the public may exhibit differentiated time delays in policy acceptance and execution due to their inherent operational characteristics, these factors will not impede their optimal strategy selection.

Our study establishes an evolutionary game-theoretic model based on perfect rationality and expected utility theory, incorporating time delay factors to investigate strategic interactions among game entities within the medical industry's carbon neutrality context. Our analysis examines explicitly how time delays influence the strategic decision-making processes of various stakeholders. However, real-world scenarios suggest that, beyond time delays, subjective factors such as emotional states and risk attitudes significantly impact agents' strategic choices. These psychological dimensions can induce stochastic mutations during evolutionary processes, potentially accelerating convergence to optimal strategies. Consequently, future research could extend this model by integrating stochastic perturbation terms and implementing multi-stage repeated game analyses.

## Data Availability

The original contributions presented in the study are included in the article/supplementary material, further inquiries can be directed to the corresponding author.
